# Drivers of Perceived Nuisance Growth by Aquatic Plants

**DOI:** 10.1007/s00267-022-01781-x

**Published:** 2023-01-11

**Authors:** Kirstine Thiemer, Bart Immerzeel, Susanne Schneider, Keneilwe Sebola, Julie Coetzee, Mathieu Baldo, Gabrielle Thiebaut, Sabine Hilt, Jan Köhler, Sarah Faye Harpenslager, Jan E. Vermaat

**Affiliations:** 1 Section for Nature based Solutions and Aquatic Ecology, Norwegian Institute for Water Research, Økernveien 94, 0579 Oslo, Norway; 2 Faculty of Environmental Sciences and Natural Resource Management, Norwegian University of Life Sciences, P.O. Box 5003, 1430 Ås, Norway; 3 Centre for Biological Control, Botany Department, Rhodes University, PO Box 94, Grahamstown, 610 South Africa; 4 Université de Rennes 1, Campus Beaulieu, UMR 6553 CNRS ECOBIO, 263 Avenue du Général Leclerc, 35042 Rennes, France; 5 Dept. of Community and Ecosystem Ecology, Leibniz Institute of Freshwater Ecology and Inland Fisheries, Müggelseedamm 301, 12587 Berlin, Germany; 6 Present Address: Norwegian Institute for Nature Research, Sognsveien 68, 0855 Oslo, Norway; 7 Present Address: B-Ware Research Centre, Postbus 6558, 6503 GB, Nijmegen, The Netherlands

**Keywords:** Macrophytes, mass development, ecosystem services, survey, problematic growth, Bayesian networks

## Abstract

Mass developments of macrophytes occur frequently worldwide and are often considered a nuisance when interfering with human activities. It is crucial to understand the drivers of this perception if we are to develop effective management strategies for ecosystems with macrophyte mass developments. Using a comprehensive survey spanning five sites with different macrophyte species in four countries (Norway, France, Germany and South Africa), we quantified the perception of macrophyte growth as a nuisance among residents and visitors, and for different recreational activities (swimming, boating, angling, appreciation of biodiversity, appreciation of landscape and birdwatching). We then used a Bayesian network approach to integrate the perception of nuisance with the consequences of plant removal. From the 1234 responses collected from the five sites, a range of 73–93% of the respondents across the sites considered macrophyte growth a nuisance at each site. Residents perceived macrophytes up to 23% more problematic than visitors. Environmental mindedness of respondents did not influence the perception of nuisance. Perceived nuisance of macrophytes was relatively similar for different recreational activities that were possible in each case study site, although we found some site-specific variation. Finally, we illustrate how Bayesian networks can be used to choose the best management option by balancing people’s perception of macrophyte growth with the potential consequences of macrophyte removal.

## Highlights

- Aquatic plants that hinder human activities are perceived as a nuisance
- We identified drivers of perceived nuisance at five sites with dense aquatic vegetation
- Visitors were less likely to consider macrophytes a nuisance than residents
- Type of recreational activity affected perceived nuisance but differences were small

## Introduction

Freshwater ecosystems make up only 0.01% of the world’s water (Dudgeon et al. 2006), yet this small fraction constitutes highly valuable natural resources from which human societies receive important ecosystem services (i.e., human benefits obtained from nature) (Janssen et al. 2021). Aquatic macrophytes are considered vital in freshwater ecosystems as their presence influences both physical, chemical and biological characteristics of aquatic ecosystems (Jeppesen et al. 1998) and consequently, a series of ecosystem services (Grizzetti et al. 2016; Janssen et al. 2021; Millennium Ecosystem Assessment 2005). Ecosystem services provided by macrophytes include supporting (habitats for periphyton, invertebrates and fish), provisioning (food, fertiliser, biomass fuel), regulating (carbon sequestration, nutrient retention, water purification, pest and disease control) and cultural services (recreation activities, appreciation of landscape and appreciation of biodiversity non-use) (Boerema et al. 2014; Janssen et al. 2021). The societal benefits that macrophytes provide may, however, be diminished when macrophytes occur at high densities (i.e., mass development), as macrophytes are often perceived a nuisance when they impede drainage (Baattrup‐Pedersen et al. 2018), irrigation (Armellina et al. 1996) or recreational activities (Verhofstad and Bakker 2019).

In freshwater ecosystems, solutions to combat this perceived nuisance growth include mechanical removal (cutting and dredging), chemical control (herbicides) and biological control (herbivorous fish, manatees or insects), where mechanical removal is the most common management practice in the Northern hemisphere (Hilt et al. 2006; Vereecken et al. 2006; Verhofstad and Bakker 2019). Mechanical macrophyte removal may eliminate nuisance macrophyte growth and thereby reduce the interference of macrophytes with human activities (Verhofstad and Bakker 2019). Freshwater managers should seek to reach a macrophyte growth level that maximises the total ecosystem services value (Janssen et al. 2021) which may involve balancing people’s perception and consequences of removal for the ecosystems. Bayesian networks (BNs) have previously been used by water managers as a decision support tool (Langmead et al. 2009; Stewart‐Koster et al. 2010), and may be useful to integrate different user groups’ perceptions of macrophyte growth and the consequences of macrophyte removal on ecosystem properties, assisting water managers in optimizing their strategies. A Bayesian network is a model based on probabilities, which can be constructed from a system of boxes (parent and child nodes) connected by arrows that represent conditional dependencies, each with a probability (Stewart-Koster et al. 2010). The network is quantified by conditional probability tables (CPTs) for each child node that can be quantified either by observational data or expert knowledge (Korb and Nicholson 2004; Pollino et al. 2007). Water managers can manipulate the BN to simulate the effects of different management scenarios on ecosystem services. For the BN to deliver reliable probabilities, information is required on the consequences of different removal alternatives and information on when macrophytes become a nuisance to optimise the management of ecosystems with mass developments.

Although many individual studies have quantified the consequences of macrophytes removal (reviewed by Thiemer et al. (2021)) and nuisance growth of macrophytes has been regularly reported in scientific reports and popular media, only one study reports on perception of submerged macrophytes as a nuisance (Verhofstad and Bakker 2019). Perceived nuisance from free-floating and emergent macrophytes remains largely unexplored. Perception of macrophytes as nuisance is likely to depend on different parameters such as the spatial extent of the vegetation, the species (including the notion of invasiveness), plant life-form (submerged, free-floating or emergent), type of activity (swimming, boating, angling etc.) and socio-demographic parameters (resident/visitor, environmental-mindedness). Correspondingly, Verhofstad and Bakker (2019) concluded that creating a single threshold for cover and clear water depth above the macrophyte canopy is impossible and that classification of nuisance levels will benefit from including site-specific information on the perception of nuisance.

Building on this lack of quantitative data, we explored the level at which macrophytes are perceived as nuisance and the patterns in underlying drivers. We carried out a survey among residents and visitors in five study sites. To allow for comparative analysis, the surveys had the same design and number of questions but differed in the specification of the local macrophyte mass development problem (Table 1). We expect that: (i) higher abundance or cover of macrophytes will cause a higher probability of perceived nuisance; (ii) nuisance thresholds vary between respondent type (resident and visitor), where residents may perceive macrophytes as nuisance at lower levels since they are more likely to be frequently exposed to the macrophytes and to have a priori knowledge of the nuisance issue and removal practices at the given site, which visitors do not necessarily have; (iii) respondents with higher environmental mindedness will consider macrophyte growth less of a nuisance; and (iv) nuisance thresholds are influenced by respondent activities, where perceived nuisance is likely to be higher for recreational activities such as swimming, boating and angling compared to appreciation of biodiversity, appreciation of landscape and birdwatching. We expanded the BN of Thiemer et al. (2021) to illustrate how it is possible to integrate user perceptions of mass developments with the possible effects of different management options. Such BN can be developed into a management decision support tool that can optimise the management of ecosystems with macrophyte mass developments.

**Table 1:** Socio-demographic profiles of respondents for the five study sites

Characteristics	*E. nuttallii*	*J. bulbosus*	*Ludwigia*	*P. crassipes*	*S. sagittifolia*
Country	Germany	Norway	France	South Africa	Germany
Ecosystem	Lake Kemnade	River Otra	Lake Grand-Lieu	Lake Hartbeespoort Dam	River Spree
Species status	Non-native	Native	Non-native	Non-native	Native
Coordinates (lat,long)	51.424439, −7.266353	59.088090, 7.551812	47.098278, −1.662513	−25.749746, 27.854470	52.383337, 13.953934
*N* (% remaining)	292 (73%)	172 (42%)	304 (89%)	299 (63%)	167 (63%)
Total N	403	297	338	477	265
Online/on-site	164/128	91/81	68/236	255/44	167/0
Age (median)	45.9 (17.1)	45.8 (15.2)	45.5 (16.6)	48.8 (14.7)	47.2 (14.4)
Female (%)	42%	46%	33%	39%	38%
Visitors (%)	74%	58%	35%	21%	34%
Mean NEP-score (1–5)	3.9 (0.5)	3.4 (0.6)	3.6 (0.5)	3.7 (0.5)	3.9 (0.6)
Time of collection (Both on-site and Online surveys)	July–August 2020	June–September 2020	July–August 2020	January 2020	June–August 2020

Median/mean with standard deviation in brackets for the main characteristics of respondents for each study area. NEP-scores range from 1 to 5 with low numbers indicating anthropocentric and high number ecocentric opinions.

## Methods

### Perception of Macrophyte Growth

We used surveys to obtain data on people’s perceptions of macrophyte growth in relation to different user activities in five different study sites: Lake Kemnade in Germany, dominated by invasive *Elodea nuttallii* ((Planch) St. John), Hartbeespoort Dam in South Africa, dominated by invasive *Pontederia crassipes* (Mart.), Lake Grand-Lieu in France, dominated by invasive *Ludwigia* species, River Otra in Norway, dominated by the native *Juncus bulbosus* (L.) and River Spree in Germany, dominated by several native macrophytes (mainly *Sagittaria sagittifolia* (L.)) (Table 1).

**Fig. 1:**
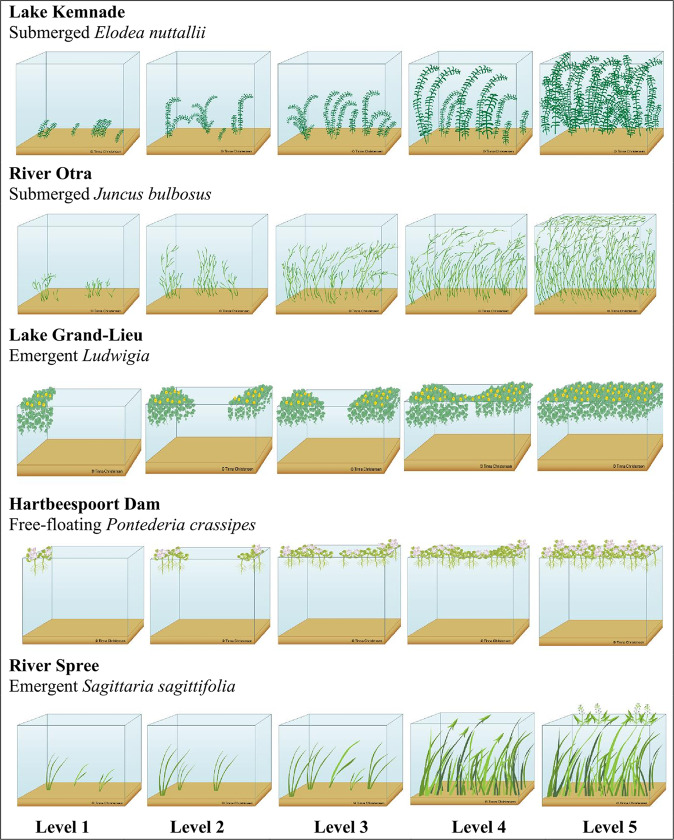
Pictures used in the survey question assessing the perceived nuisance level for each of the five macrophytes species at five case study sites. The five study sites were Lake Kemnade in Germany dominated by the invasive *Elodea nuttallii*, the River Otra in Norway dominated by the native *Juncus bulbosus*, Lake Grand-Lieu in France dominated by invasive *Ludwigia* species, Hartbeespoort Dam in South Africa dominated by invasive *Pontederia crassipes*, and the River Spree in Germany dominated by several native macrophytes (mainly *Sagittaria sagittifolia*)

The surveys used stylised images of different levels of aquatic plants. Choice experiments and willingness-to-pay surveys often use artwork of varying schematisation (e.g., Bateman et al., 2011 versus Immerzeel et al. 2022). A good reason for using stylized artwork rather than photographs is that stylized artwork lacks potentially confounding aspects often common in photographs, such as sun angle (an issue with water plants), cloudiness, or distracting features. In addition, our survey focused on different levels of aquatic plant density for different forms of use (or non-use). By using stylised images of different levels of aquatic plants, the respondents were invited to imagine how these different levels would impact their use of the lake/river and if it would be considered a nuisance.The surveys had a common structure (see Supplementary Information 1 for an example) but were adjusted to local conditions (i.e., swimming is not allowed in Lake Kemnade and Lake Grand-Lieu, and birdwatching was included as a separate activity only in Grand-Lieu). To classify the perception of macrophyte growth, respondents were asked to choose level(s) of macrophyte growth (ranging from 1–5) that they considered a nuisance (Fig. 1, i.e., not ticking a level was considered as answering not a nuisance). In addition, respondents were asked to distribute 100 points across 4–5 activities (swimming, boating, angling, appreciation of biodiversity, birdwatching and appreciation of landscape) as an indicator of the importance of each of these activities for the individual respondent. The last part of the survey covered a sequence of questions on general social-demographic information including age and gender that was used to give context. Validation of sample representativeness was not performed, as these surveys were not designed to represent the whole population. We included questions on what the respondents’ decisions on levels for nuisance were based, as well as a standard set of questions targeting a respondent’s opinion on environmental issues. For this purpose. we included the New Environmental Paradigm Scale (hereafter NEP-score), which has been developed to estimate the environmental-mindedness of the respondents’ worldview (Dunlap 2008; Dunlap et al. 2000; Dunlap and Van Liere 1978). To calculate the NEP, the respondents were presented a series of statements that either support an anthropogenic or ecocentric world view (Immerzeel et al. 2022) and respondents rated to what degree they agreed on the statements on a scale from “strongly disagree” to “strongly agree”. The NEP-score was calculated for each respondent by transforming the responses “Strongly disagree” to “Strongly agree” into a 1–5 scale and then calculating the arithmetic mean across all the questions, as described in Dunlap et al. (2000). A low NEP-score means a more anthropogenic worldview whereas a larger NEP-score a more ecocentric worldview. An example of the surveys from the River Otra can be retrieved in Supplementary Information 1.

### Data Collection

Prior to data collection, the surveys were translated into local language by native speakers. An English version was available in all study sites for respondents not speaking the local language. The surveys were qualitatively pre-tested twice for each study site. Pre-testing included a variety of scientists (not involved in this study) reading through and commenting on the survey, and we also distributed the surveys among friends and families to do the same. A second round of pre-testing with the same pre-testers was performed after including the suggestions received from the first pre-testing round. Quantitative pre-testing on a sub-sample of the population, as suggested by Johnston et al. (2017), was not possible, due to the limited time budget and the large geographic spread of the study sites.

We collected the surveys for the five study sites using both an online version and a printed version. We targeted respondents that were using the area defined in a map within each survey. The combination of on-site and online surveys helped in achieving the required sample size of 150 responses per study area and likely enhanced representativeness by covering a broader suite of respondents, as using only on-site collected surveys could introduce a sampling bias (Lindhjem and Navrud 2011). The online versions were distributed via e-mail lists, social media, websites for local organisations and hand-out QR codes. The printed version was collected from on-site encounters and pick-up and drop-off places. At each study site, 2–6 surveyors visited the area and distributed printed surveys among respondents at local recreation hotspots, shops, museums, tourist visitor centres and other public areas. The surveys on-site were distributed throughout the summer months (growing season of aquatic macrophytes) and included both weekdays, weekends and public holidays. The online survey was promoted in the same time frame as the on-site survey. The on-site collection of the printed surveys was done in accordance with the Covid-19 restrictions prevalent at the given time for each site. Our surveys were anonymous and complied with the data protection and privacy rules in the given country.

### Data Preparation

Survey data are prone to various types of selection bias (Johnston et al. 2017), thus prior to the analyses we checked the survey data, by removing non-response answers and inaccurate or clearly inconsistent “protest” answers (for example, distributing more than 100 points when asked to distribute 100 points) (Pennington et al. 2017). We used a conservative strategy to remove responses and only included respondents that filled out the willingness to pay questions (a criterion needed for the study on economic valuation of ecosystem services by Vermaat et al. in prep. which uses the same case study sites and build on results of this study). Consequently, between 42 and 89% of the collected surveys could be used, depending on the case study site. The question regarding perceived nuisance was mistranslated for the survey on *Ludwigia*, where respondents were asked to indicate the lowest level at which they perceived the growth a nuisance, hence leaving higher levels unticked. The answers were adjusted for these respondents by giving all above levels from the ticked level the value 1 (i.e., nuisance).

## Data Analyses

All statistical analyses were made in R version 3.6.4 (R Core Team 2020) using the following packages: lme4 (Bates et al. 2021), emmeans (Lenth et al. 2022) and MASS (Ripley et al. 2021). Graphics were made using the R package ggplot2 (Wickham et al. 2020). The Bayesian networks were built using the NETICA software v. 6.07 (Norsys 2005).

### Perception of Macrophyte Growth

Perception of macrophyte growth (i.e., probability of perceiving growth as a nuisance) was analysed using generalized linear mixed models (GLMMs) with a binomial family (log-link). This analysis was carried out separately for each of the five sites, as the macrophyte growth levels (pictures 1–5 from surveys, used as continuous predictor, Fig. 1) only correspond qualitatively among the species, but do not reflect the same absolute biomass or plant density.

Initially, the influence of macrophyte growth level (1–5), respondent type (resident, visitor) and ecological mindedness of respondents (NEP-score) on the probability of perceiving macrophytes as a nuisance (0 or 1) were examined. Candidate models with the interaction between respondent type and macrophyte growth level were compared to models without the interaction using Akaike information criteria (AIC), in which the most strongly supported model has the lowest AIC (Anderson 2007). When the difference in AIC among two models (delta AIC) was lower than 2, the simplest model was chosen (Burnham and Anderson 2004). Respondent IDs were set as a random effect to account for the lack of independence of observations made by each respondent, i.e., to cope with covariance among answers from individual respondents. Macrophyte growth levels at which the probability for perceived nuisance was 50%, hereafter called median nuisance levels, were estimated using the *dose.p* function from the MASS package (Ripley et al. 2021).

To understand how macrophyte growth is perceived by respondents when engaged in different activities, the influence of activity on the probability of nuisance were tested using GLMMs with a binomial family (log-link) for each of the five sites separately, as not all activities were possible for the respondents at the respective site. Interaction between activity and respondent type, activity and macrophyte growth, respondent type and macrophyte growth were tested by comparing candidate models with and without these interactions and selecting the model with the lowest AIC (Anderson 2007). All observations (Nuisance 0 or 1) were weighted by the proportion of the 100 points from the question on the importance of activities for each respondent, to differentiate between respondents with a clear preference for one activity, and respondents with a more “casual” use of the ecosystem for several activities.

### Decision Support Tool for Water Managers Using Bayesian Network Approach

We used the BN approach as a first attempt to build a decision support tool for water managers in charge of ecosystems with macrophyte mass developments, by integrating people’s perceptions of macrophytes and the short-term consequences of mechanical macrophyte removal. The CPTs in the BNs are based on empirical perception patterns from the surveys (probabilities of nuisance perception for combinations of respondent type, activity, macrophyte species and macrophyte growth levels). They are here integrated with an existing BN on short-term consequences of mechanical removal developed by Thiemer et al. (2021), which was based on qualitative, limnological expert knowledge for illustrative purpose. A detailed description of the network can be found in Supplementary Information 2.

### Description of the Decision Support Tool (Bayesian Network)

**Fig. 2:**
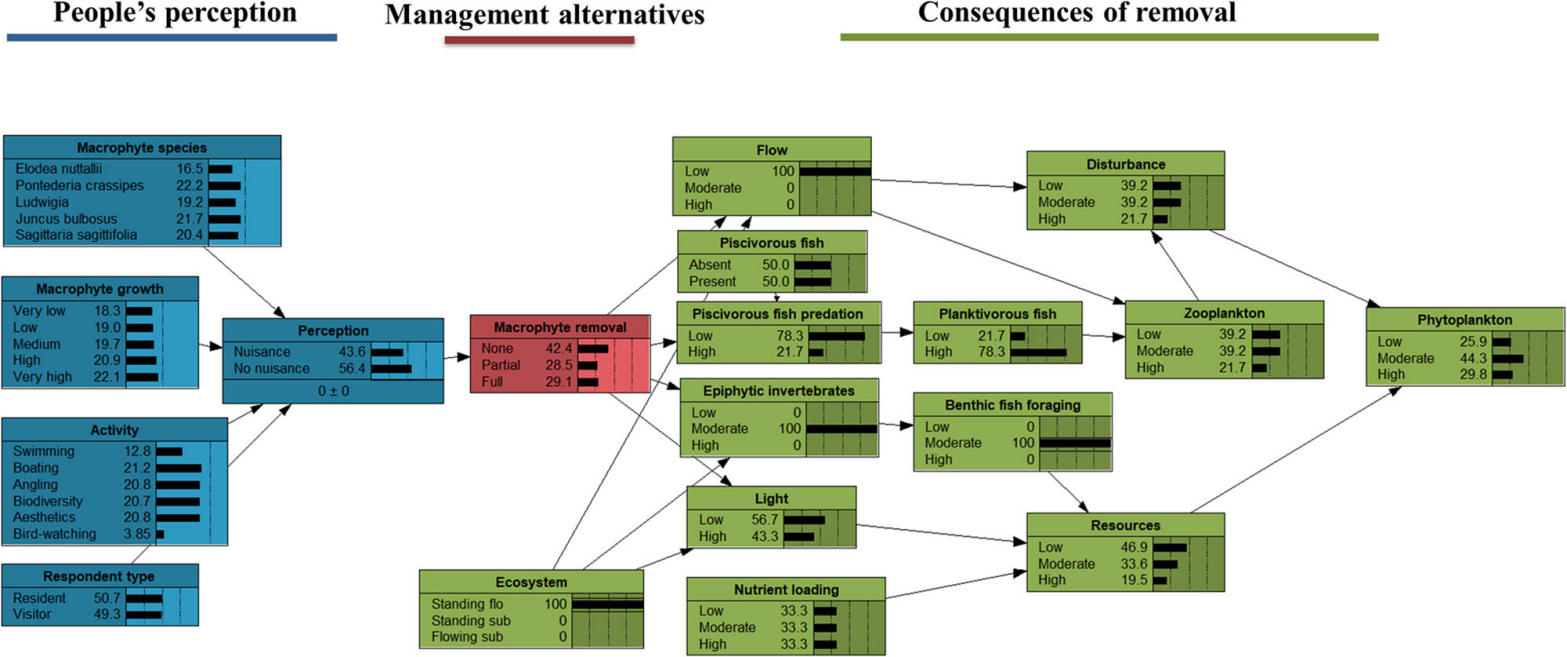
BN integrating people’s perception of macrophyte growth, consequences of macrophyte removal and potential management alternatives. All nodes were linked with conditional probability tables (Supplementary Information 2). Nodes were characterised by their states (1–5). The BN component on perception builds on the currently presented survey data whereas the component on consequences is taken from Thiemer et al. (2021) where CPTs were based on expert knowledge derived from the literature

In the part of the BN quantifying people’s perception of macrophyte growth (Fig. 2), *Perception* is a function of four predictor variables *Activity, Respondent type, Macrophyte species* and *Macrophyte growth level* which are all likely to influence the perception of people. Since environmental mindedness was not significant in the GLMM analyses, we left it out of the model. *Plant management option* indicates the proportion of macrophyte removal. *Plant management option* links the *People’s perception* with the BN on short-term consequences of macrophyte removal developed by Thiemer et al. (2021). This part of the BN illustrates the short-term effect of macrophyte removal on ecosystem structure with a focus on a food web model (*Phytoplankton* as end-point), because one major consequence of cutting aquatic plants is the increased risk of phytoplankton blooms (Kuiper et al. 2017). *Phytoplankton* growth is controlled by *resources (Light* and *Nutrient availability)* and *disturbances* (*Flow* and *Trophic cascade*) (Bernes et al. 2015; Reynolds 2000) and can be adjusted to local conditions changing the nodes *Ecosystem* and *Nutrient loading*. In this BN, water managers can either set the risk of a phytoplankton bloom (endpoint) to a specific target and see how probabilities are affected backwards throughout the whole BN, identifying key nodes on which the set target depends, or set the target group of people (e.g. residents’ angle) and see which management alternative is recommended and what the consequences for the ecosystem will be. Finally, setting both a target for a specific user group and for ecosystem properties is possible with the BN. This will allow for a systematic evaluation of management alternatives.

## Results

### Perception of Macrophyte Growth

A total of 1234 survey responses were retained after quality control and analysed, with sample sizes varying between 167 and 304 for the five study areas (Table 1). Overall, the fraction of respondents considering at least one of the macrophyte growth levels a nuisance was high, ranging from 70–99% and 66–95% for residents and visitors, respectively, across the five sites (Supplementary Information, 2, Fig. S2). The fraction of respondents answering “I don’t know” was higher for visitors (2–34%) than for residents (1–8%, Supplementary Information, Fig. S2).

**Fig. 3:**
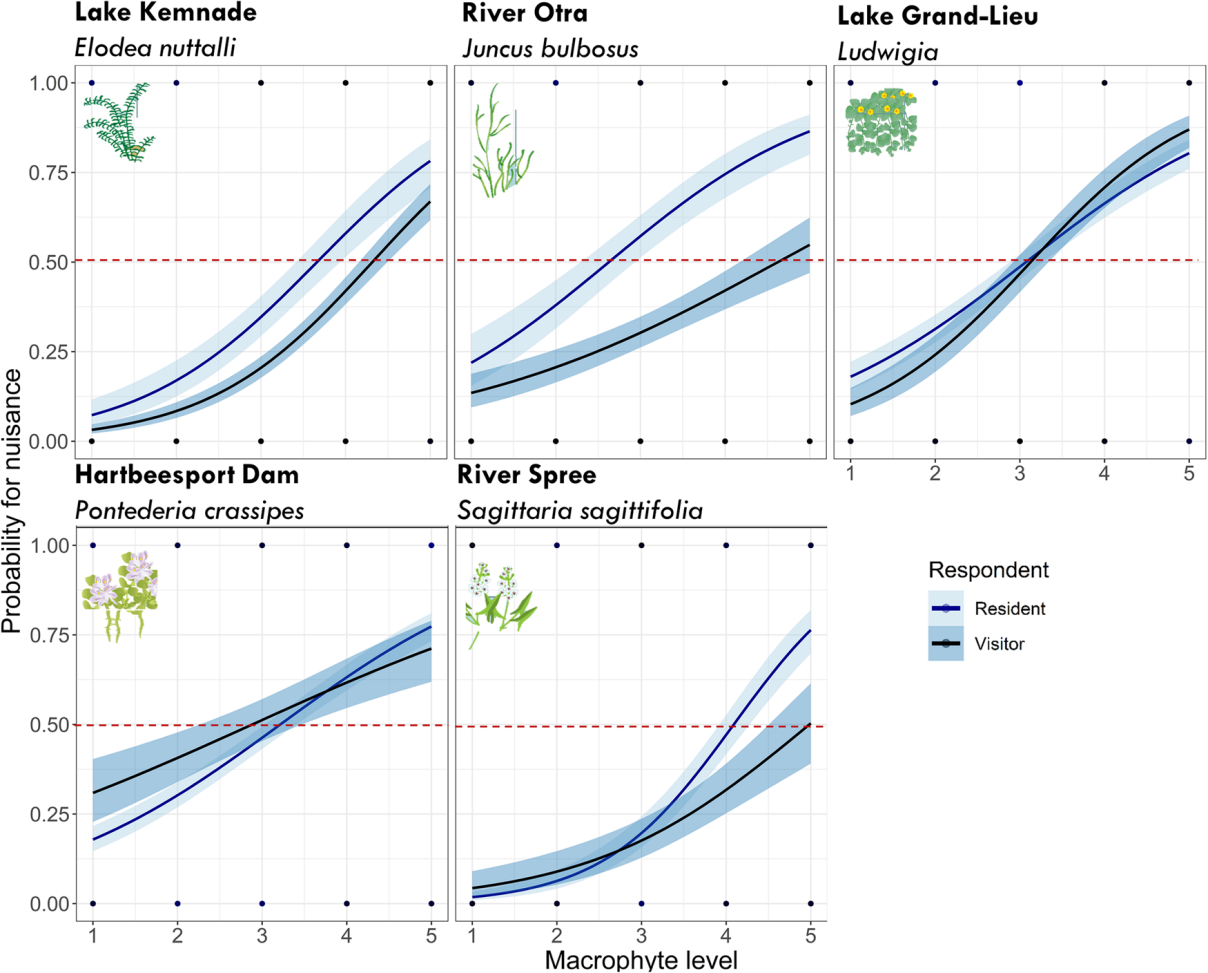
Probability of perceived nuisance in relation to macrophyte growth level (1–5), macrophyte species and respondent types (resident and visitor) (GLMMs). Bands are confidence intervals (0.95). The red dashed line represents the level at which probability of nuisance is 50%

**Fig. 4:**
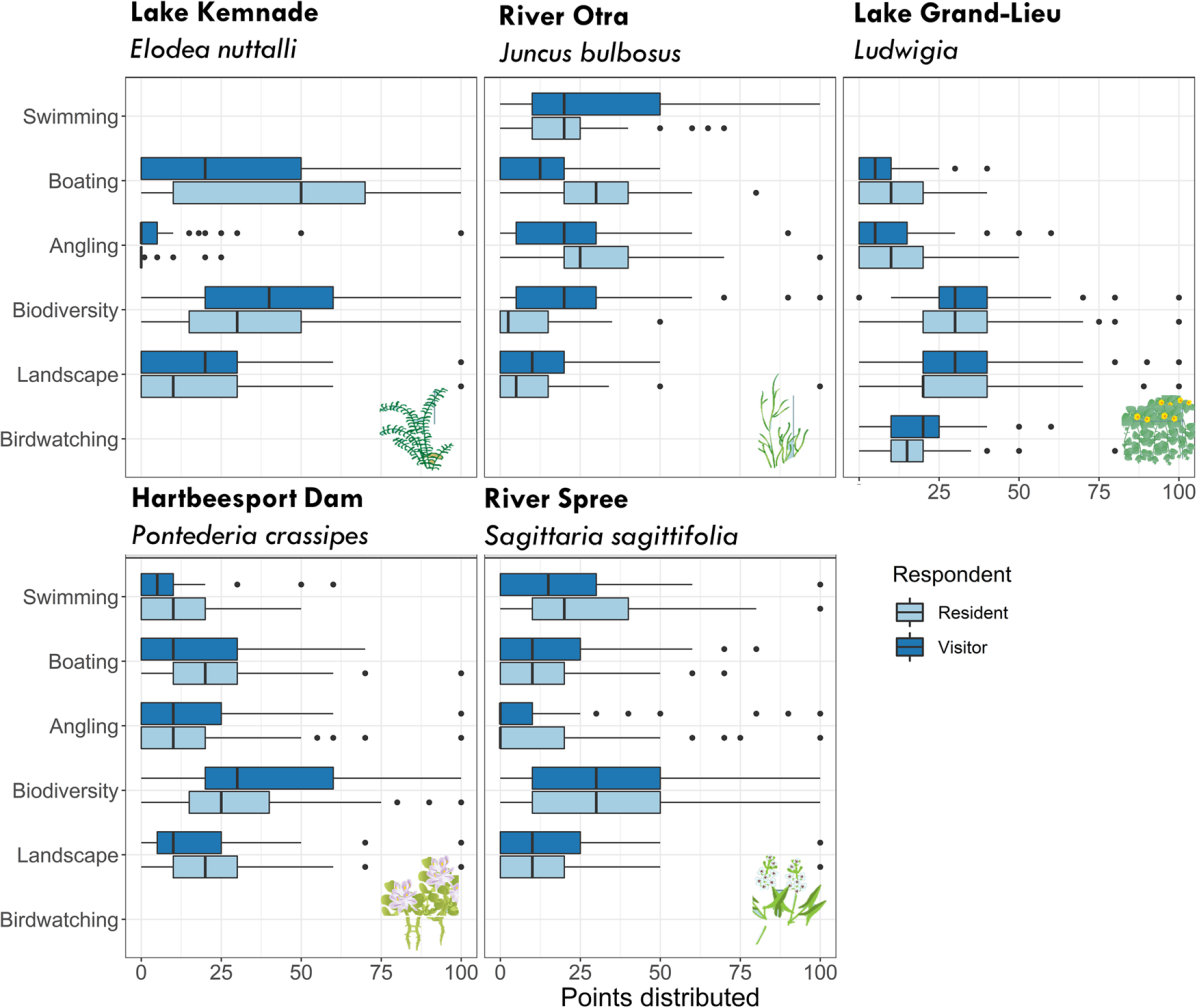
Boxplot showing the distribution of points (0–100) given by respondents to different activities at the five sites characterised by different macrophyte species (for residents and visitors, respectively). Vertical bold lines indicate the median, boxes the 25% and 75% percentiles, and whiskers the minimum and maximum values

For all macrophyte species, the probability that macrophytes were perceived as a nuisance increased with macrophyte growth level (Fig. 3, Supplementary Information 3, Table S1). *E. nuttallii* and *S. sagittifolia* had considerably lower probabilities for perceived nuisance at low macrophyte growth levels (<3) than the other three species (Fig. 4). A comparison of the median perceived nuisance levels among the five species (Fig. 4) shows that *Ludwigia* spp. (3.1 ± 0.1 SD) and *P. crassipes* (3.2 ± 0.1 SD) were considered a nuisance already at low levels followed by *J. bulbosus* (3.6 ± 0.1 SD), *E. nuttallii* (4.1 ± 0.1 SD) and *S. sagittifolia* (4.3 ± 0.1 SD). Visitors generally had a lower probability of considering growth of *E. nuttallii* and *J. bulbosus* a nuisance than residents (Fig. 3, Supplementary Information 3, Table S1). This difference in probability was 24% for *J. bulbosus* and 10% for *E. nuttallii*, respectively. Visitors’ and residents’ perception did not differ for *Ludwigia* (Fig. 3, Supplementary Information 3, Table S1). Interestingly, for *S. sagittifolia* and *P. crassipes* the interaction between respondent type and macrophyte growth level was significant. This suggests that the increase in probability for nuisance with increasing macrophyte growth was not the same for visitors and residents. For *S. sagittifolia*, the two probability curves are parallel at lower plant levels but start to deviate at higher plant levels, whereas the opposite was found for *P. crassipes* (Fig. 3). Finally, the environmental mindedness of the respondents (NEP-score) did not influence the perception of macrophytes as a nuisance (GLMMs, Supplementary Information 3, Table S1).

### Perception of Macrophyte Growth among Different Activities

**Fig. 5:**
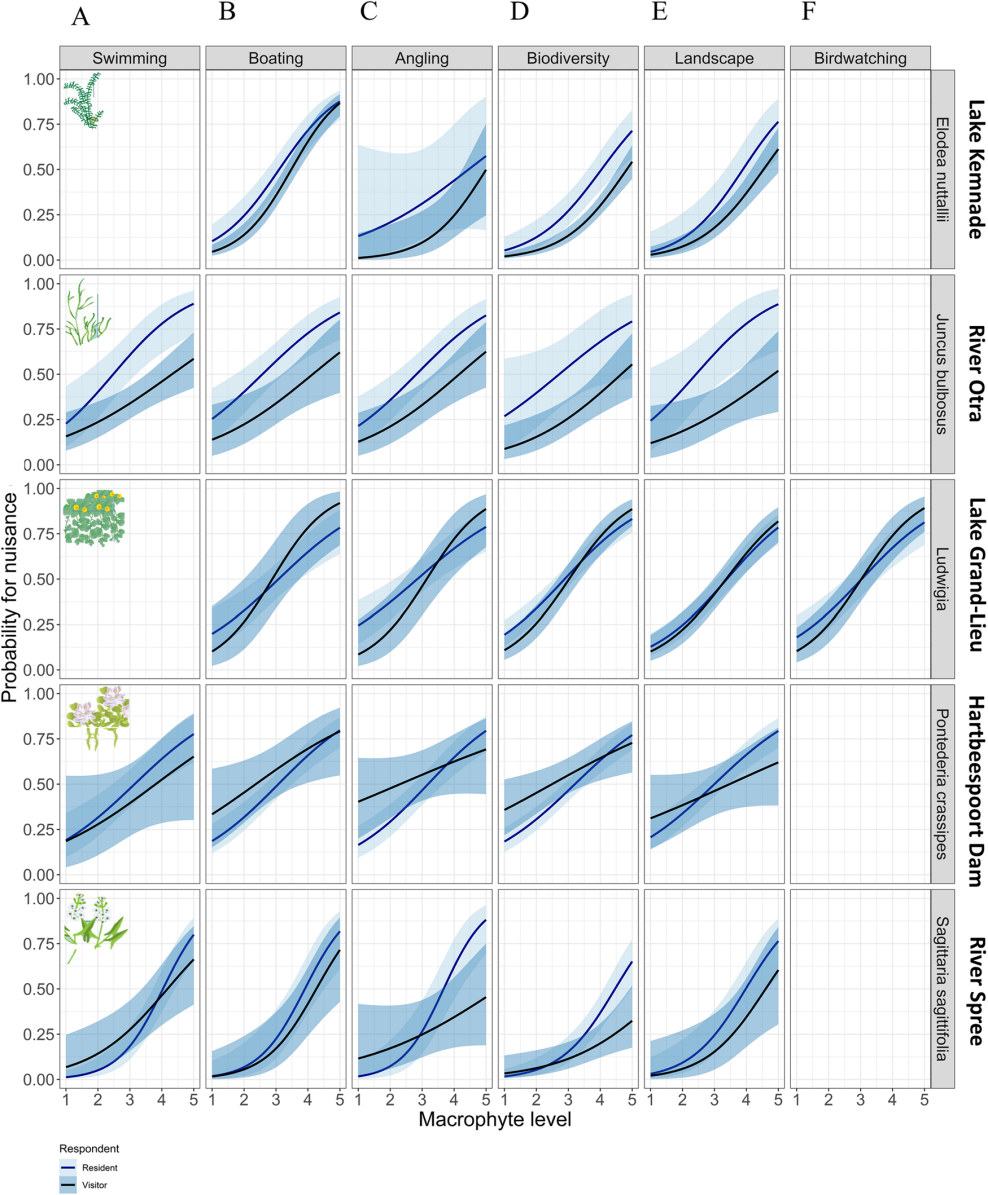
Probability of perceiving macrophytes as a nuisance with increasing macrophyte growth level, between macrophyte species and respondents for six activities (**A**) Swimming, (**B**) Boating, (**C**) Angling, (**D**) Appreciation of biodiversity, (**E**) Appreciation of landscape, (**F**) Birdwatching. Bands are confidence intervals (0.95). Note that not all activities were present in all case studies

Preferred activities of individual respondents were obtained from the question where respondents could distribute 100 points among four to six (dependent on the study site) activities. This distribution of points revealed that most respondents were engaged in more than one activity and only few respondents gave all 100 points to a single activity (Fig. 4). Overall, the activity type had a significant effect on the level of perceived nuisance, yet macrophyte growth level, respondent type and their interaction explained most variation in perceived nuisance (Fig. 5, Table 2). Different patterns in probability for perceived nuisance in relation to preferred activity were found for the five macrophyte species, regardless of their interaction with respondent type and the interaction of respondent type and macrophyte growth level (Fig. 5A–F). The probability of perceiving the submerged *J. bulbosus* growth as a nuisance was in general high for all activities (i.e., user groups), yet the probability of perceived nuisance was 41% (±15%, SE) higher for respondents who stated swimming as an important activity compared to respondents stating that appreciation of landscape was important, when controlling for all other variables (Fig. 5). Finally, a significant interaction of respondent type and activity was found for *P. crassipes*, suggesting that visitors and residents did not equally consider growth of *P. crassipes* a nuisance with increasing macrophyte growth between different activities (Fig. 5). For *Ludwigia* in Lake Grand-Lieu, activity type had no significant effect on the level of perceived nuisance.

**Table 2:** GLMMs results for probability of perceiving macrophyte as a nuisance in relation to activity type, respondent type and macrophyte growth

	Variable	Chisq	Df	*P*
**Lake Kemnade** *E. nuttallii*				
	Activity	79.5	3	**<0.0001**
	Respondent	2.37	1	0.124
	Macrophyte growth	131.68	1	**<0.0001**
**River Otra** *J. bulbosus*				
	Activity	7.93	4	**0.094**
	Respondent	2.25	1	0.133
	Macrophyte growth	9.23	1	**0.002**
**Lake Grand-Lieu** *Ludwigia*	Activity	18.99	4	**0.001**
	Respondent	5.27	1	**0.021**
	Macrophyte growth	8.19	1	**<0.001**
**Hartbeespoort Dam** *P. crassipes*				
	Activity	19.39	4	**0.001**
	Respondent	4.31	1	0.038
	Macrophyte growth	166.7	1	**<0.001**
	Activity: Respondent	26.4	4	**<0.001**
	Respondent: macrophyte growth	13.42	1	**0.001**
**River Spree** *S. sagittifolia*	Activity	13.96	4	**0.007**
	Respondent	5.67	1	0.017
	Macrophyte growth	28.06	1	<**0.001**
	Respondent: macrophyte growth	4.92	1	**0.026**

Bold *P* values represent significant levels at 0.05.

*Df* degrees of freedom.

### Identifying best Management Alternatives for Different user Groups

**Fig. 6:**
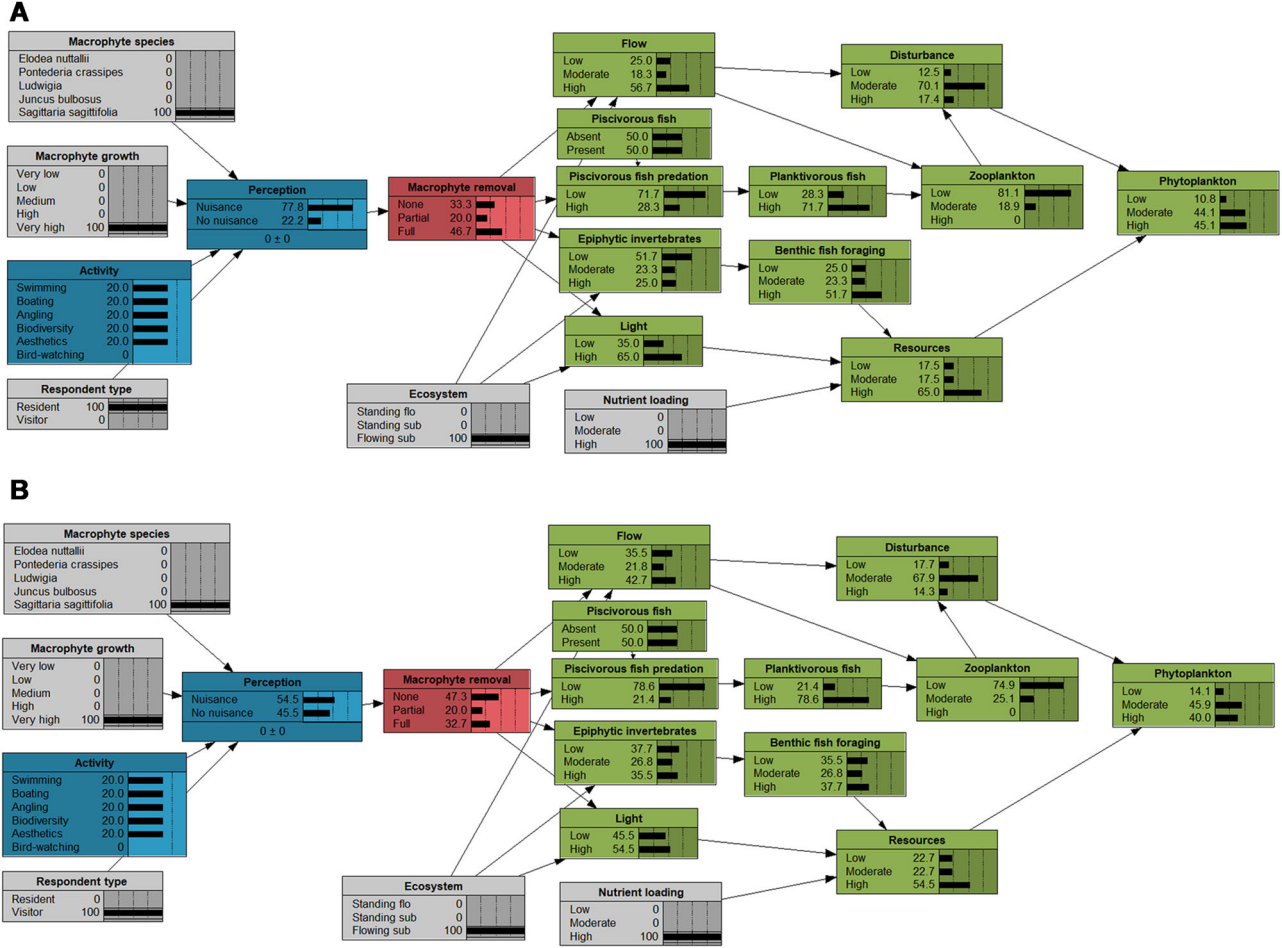
BNs of probability of management alternatives for a riverine system with high nutrient loading and very high *S. sagittifolia* growth (**A**) probabilities for respondent type is set to resident (**B**) probabilities for respondent type is set to visitors. Grey boxes indicate nodes that have been specified

In the following two examples, the BN is adjusted to a hypothetical freshwater river that has high nutrient loadings and experiences mass development of the emergent species *S. sagittifolia*, by setting the probabilities to 100% of the states for the respective nodes (*macrophyte species, macrophyte growth* and *nutrient loading*) (Fig. 6A). If we assume that the users of this ecosystem only consist of residents (*respondent type* set to 100% residents), the probability for a respondent to perceive high growth of *S. sagittifolia* a nuisance will then be 77.7% and the BN then suggests that the best management option would be full removal (probability for this option was 46.7%) (Fig. 6A). By only changing the respondent type from resident to visitor, this probability of perceived nuisance decreases to 54.5% and the suggested management option would now be no removal (Fig. 6B). The impact of the two different removal alternatives on the probability of high phytoplankton concentrations are considerable, where choosing full removal over no removal would result in an increased probability of algal blooms (state of high phytoplankton abundance) from 25% to 63% (Supplementary Information 2, Figs. S3 and S4).

Assuming the same conditions as in the previous example, a goal for managers could be to manage the mass development for specific user groups, for instance anglers, boaters, swimmers or people appreciating biodiversity. By setting the activity to boating, the perception of nuisance and management alternative suggested is full removal (probability for this option was 48%), whereas changing the activity to appreciation of biodiversity, no removal is suggested (probability for this option was 40%) (Supplementary Information 2, Figs. S5 and S6).

## Discussion

Our results supported the hypothesis that an increasing extent of macrophyte growth resulted in a higher probability of perceived nuisance, but differences occurred among the investigated macrophyte species and/or sites. As expected, nuisance thresholds were influenced by respondent activities and varied between respondent type with residents perceiving macrophytes a nuisance at lower levels than visitors. Contrary to our expectation, respondents with a higher ecological mindedness did not consider macrophyte growth less of a nuisance. We show that integrating this knowledge on user perceptions into a Bayesian network-based decision support tool can optimise the management of macrophyte mass developments.

### Identifying Drivers for Perceiving Macrophyte Growth as Nuisance

The probability of perceiving macrophyte growth a nuisance was related to macrophyte growth level, with higher macrophyte growth levels resulting in higher probability for perceived nuisance Visitors were up to 23% less likely to consider macrophyte growth a nuisance than residents and this difference was significant for *E. nuttallii, J. bulbosus* and *S. sagittifolia*. In general, visitors often pay shorter visits to the area and may not necessarily know the local problems with macrophytes. They may therefore not find the macrophyte growth a particular problem, which is supported by the higher proportion of visitors answering: “I don’t know” to the question on which macrophyte levels they considered as nuisance growth compared to residents (visitors: 2–37%, residents:1–8%, Fig. 3). Perception of nuisance by *Ludwigia* spp. *w*as not different among visitors and residents. It was also expected that a high environmental-mindedness (high NEP-scores) would affect the perception and include acceptance of denser macrophyte beds. However, the NEP-score had no significant effect at all. The mean NEP-scores were similar across the five sites, ranging from 3.4–3.8, where Norwegian respondents scored comparatively low and Germans high. Correspondingly, NEP-scores reported from Norway ranged between 3.5 (Immerzeel et al. 2022) and 3.8 (Bjerke et al. 2006) and for Germany between 4.1 and 4.2 (Kaiser et al. 2005; Schultz et al. 2005). Overall, the currently observed NEP-scores fall within the expected range (3.8 ± 0.3 SD) from a meta-analysis by Hawcroft and Milfont (2010). This suggests that respondents in our five case study sites generally place an average to high value on nature and show concern about the negative impacts that human activities can have on the environment.

### Linking Nuisance Perception to Recreation Activity Type

The probability of perceived nuisance was expected to be different for each activity as well as for each case study site with different macrophyte species. In concordance with expectations, we found significant differences between activities within each case study site, but the differences were small. More interestingly, differences in perceived nuisance in relation to activity were found among sites, which indicates that perception of nuisance may not only depend on activity, but also on macrophyte species, local context and personal characteristics of the respondents. For swimmers, macrophytes are often considered a nuisance, for example when shoots entangle arms and legs (Verhofstad and Bakker 2019), which could be a more profound problem in systems with submerged than with free-floating plants. We found that submerged *J. bulbosus* had the highest probability of being considered a nuisance at low plant densities (level 1, probability for nuisance = 25%), compared to *P. crassipes* and *S. sagittifolia* that respectively had 19% and 8% probability for nuisance at these low densities. *S. sagittifolia* was considered a nuisance at higher levels (>3), which could be a result of differences in expectations for the presence of macrophytes in this river. It is likely that respondents from lowland Germany are more used to the presence of macrophytes in rivers compared to e.g. upland Norway, where macrophytes are generally less abundant in rivers (Haslam and Wolseley 1987).

For recreational boaters, macrophytes are considered a nuisance when propellers get entangled (Verhofstad and Bakker 2019) or when floating mats directly block navigation, as reported for e.g. *P. crassipes* (Habib and Yousuf 2014; Villamagna and Murphy 2010). It was therefore not surprising that *P. crassipes* had the highest probability of becoming a nuisance for boating activity at low macrophyte growth levels (level 1), followed by the submerged macrophytes *J. bulbosus, Ludwigia* spp. and *E. nuttallii*. Furthermore, high macrophyte growth is likely to increase the risk of rods and lines getting entangled in the vegetation, causing loss of catch and gear for recreational anglers (Verhofstad and Bakker 2019). For anglers, growth of *P. crassipes* and *J. bulbosus* were more likely to be perceived a nuisance at low growth levels compared to *E. nuttallii* and *S. sagittifolia*. This difference cannot be explained by plant life-form and is more likely to be a result of local conditions such as difference in type of angling (deep water, shallow water, active or passive angling). Finally, appreciation of landscape and appreciation of biodiversity have to our knowledge never been considered as aspects of recreation that can drive the perception of macrophytes. *S. sagittifolia* and *E. nuttallii* were less likely to be perceived a nuisance for people’s appreciation of biodiversity than the other three taxa—in their context.

### Management Implications

Our results show that from a management perspective it is highly relevant to know at which level macrophytes actually are perceived as a nuisance. Macrophyte removal is rather costly (Hilt et al. 2006) and at the same time, water managers also need to secure other desired ecosystem services, such as a recreation, good water quality or a healthy fish stock. These three objectives are central for the management of freshwater ecosystems with macrophyte mass developments. The BNs developed here integrate people’s perception of macrophytes with the consequences of removal and showed that management for residents may be different than management for visitors, because the latter did not mind the macrophytes as much as the former (Fig. 7A, B). Importantly, the estimated’optimal’ management for people appreciating biodiversity in systems with macrophyte mass development was not different from that for anglers or boaters, since we did not observe any differences among these categories (Fig. 6). Overall, the current BN tool can be adjusted with little effort to local conditions, because of the character of a Bayesian network. It is important to emphasise that the probabilities in the BN module dealing with the consequences (Fig. 2) by now are based on expert knowledge. Thus, for implementation on real-word cases the states of the nodes and the conditional probabilities will have to be derived for ecosystems of interest (Thiemer et al. 2021). Finally, we encourage water managers to consider using the developed management decision support tool and to include it in conversations with stakeholders in an early phase, i.e., when developing potential management alternatives that will balance people’s perception of macrophyte growth and consequences of removal for the ecosystem.

## Supplementary Information

Supporting Information II

Supporting Information I

## References

[CR1] Anderson, DR, 2007. Model based inference in the life sciences: a primer on evidence. Springer Science & Business Media.

[CR2] Armellina AD, Bezic CR, Gajardo OA, Dall’Armellina A (1996). Propagation and mechanical control of Potamogeton illinoensis Morong in irrigation canals in Argentina. J Aquat Plant Manag.

[CR3] Baattrup‐Pedersen A, Ovesen NB, Larsen SE, Andersen DK, Riis T, Kronvang B, Rasmussen JJ (2018). Evaluating effects of weed cutting on water level and ecological status in Danish lowland streams. Freshw Biol.

[CR4] Bateman, J, (2011) Text and Image A Critical Introduction to the Visual/Verbal Divide, Routledge, Pub. London. ebookISBN: 9781315773971, 10.4324/9781315773971

[CR5] Bates, D, Maechler, M, Bolker, B, Walker, S, Christensen, RHB, Singmann, H, Dai, B, Scheipl, F, Grothendieck, G, Green, P, Fox, J, Bauer, A, Pavel NK 2021. lme4: Linear Mixed-Effects Models using “Eigen” and S4.

[CR6] Bernes C, Carpenter SR, Gårdmark A, Larsson P, Persson L, Skov C, Speed JDM, Donk EV (2015). What is the influence of a reduction of planktivorous and benthivorous fish on water quality in temperate eutrophic lakes? A systematic review. Environ Evid.

[CR7] Bjerke T, Østdahl T, Thrane C, Strumse E (2006). Vegetation density of urban parks and perceived appropriateness for recreation. Urban Urban Green.

[CR8] Boerema A, Schoelynck J, Bal K, Vrebos D, Jacobs S, Staes J, Meire P (2014). Economic valuation of ecosystem services, a case study for aquatic vegetation removal in the Nete catchment (Belgium). Ecosyst Serv.

[CR9] Burnham KP, Anderson DR (2004). Multimodel Inference: Understanding AIC and BIC in Model Selection. Sociol Methods Res.

[CR10] Dudgeon D, Arthington AH, Gessner MO, Kawabata Z-I, Knowler DJ, Lévêque C, Naiman RJ, Prieur-Richard A-H, Soto D, Stiassny MLJ, Sullivan CA (2006). Freshwater biodiversity: importance, threats, status and conservation challenges. Biol Rev.

[CR11] Dunlap RE (2008). The New Environmental Paradigm Scale: From Marginality to Worldwide Use. J Environ Educ.

[CR12] Dunlap RE, Van Liere KD (1978). The “New Environmental Paradigm”. J Environ Educ.

[CR13] Dunlap RE, Van Liere KD, Mertig AG, Jones RE (2000). New trends in measuring environmental attitudes: measuring endorsement of the new ecological paradigm: a revised NEP scale. J Soc.

[CR14] Grizzetti B, Lanzanova D, Liquete C, Reynaud A, Cardoso AC (2016). Assessing water ecosystem services for water resource management. Environ Sci Policy.

[CR15] Habib S, Yousuf AR (2014). Impact of mechanical deweeding on the phytophilous macroinvertebrate community of an eutrophic lake. Environ Sci Pollut Res.

[CR16] Haslam, SM, Wolseley, PA, 1987. River plants of western Europe: the macrophytic vegetation of watercourses of the European Economic Community. Cambridge University Press.

[CR17] Hawcroft LJ, Milfont TL (2010). The use (and abuse) of the new environmental paradigm scale over the last 30 years: A meta-analysis. J Environ Psychol.

[CR18] Hilt S, Gross EM, Hupfer M, Morscheid H, Mählmann J, Melzer A, Poltz J, Sandrock S, Scharf E-M, Schneider S, van de Weyer K (2006). Restoration of submerged vegetation in shallow eutrophic lakes – A guideline and state of the art in Germany. Limnologica.

[CR19] Immerzeel B, Vermaat JE, Juutinen A, Pouta E, Artell J (2022). Appreciation of Nordic landscapes and how the bioeconomy might change that: Results from a discrete choice experiment. Land Use Policy.

[CR20] Janssen ABG, Hilt S, Kosten S, de Klein JJM, Paerl HW, Van de Waal DB (2021). Shifting states, shifting services: Linking regime shifts to changes in ecosystem services of shallow lakes. Freshw Biol.

[CR21] Jeppesen, E, Søndergaard, M, Søndergaard, M, Christoffersen, K, 1998. The structuring Role of Macrophytes in Lakes, Ecological Studies. Springer.

[CR22] Johnston RJ, Boyle KJ, Adamowic W (Vic), Bennett J, Brouwer R, Cameron TA, Hanemann WM, Hanley N, Ryan M, Scarpa R, Tourangeau R, Vossler CR (2017) Contemporary guidance for stated preference studies. J Assoc Environ Resour Econ 4:319–405. 10.1086/691697

[CR23] Kaiser FG, Hübner G, Bogner FX (2005). Contrasting the theory of planned behavior with the value-belief-norm model in explaining conservation behavior1. J Appl Soc Psychol.

[CR24] Korb, KB, Nicholson, AE, 2004. Bayesian artificial intelligence. Chapman and Hall, Coca Raton, FL.

[CR25] Kuiper JJ, Verhofstad MJJM, Louwers ELM, Bakker ES, Brederveld RJ, van Gerven LPA, Janssen ABG, de Klein JJM, Mooij WM (2017). Mowing Submerged Macrophytes in Shallow Lakes with Alternative Stable States: Battling the Good Guys?. Environ Manag.

[CR26] Langmead, O, McQuatters-Gollop, A, Mee, LD, Friedrich, J, Gilbert, AJ, Gomoiu, M-T, Jackson, EL, Knudsen, S, Minicheva, G, Todorova, V, 2009. Recovery or decline of the northwestern Black Sea: A societal choice revealed by socio-ecological modelling. Ecol. Model., Selected Papers from the Sixth European Conference on Ecological Modelling—ECEM ’07, on Challenges for ecological modelling in a changing world: Global Changes, Sustainability and Ecosystem Based Management, November 27-30, 2007, Trieste, Italy 220, 2927–2939. 10.1016/j.ecolmodel.2008.09.011

[CR27] Lenth, RV, Buerkner, P, Herve, M, Love, J, Miguez, F, Riebl, H, Singmann, H, 2022. emmeans: Estimated Marginal Means, aka Least-Squares Means.

[CR28] Lindhjem H, Navrud S (2011). Are Internet surveys an alternative to face-to-face interviews in contingent valuation?. Ecol Econ, Spec Sect - Gov Commons: Learn Field Lab Exp.

[CR29] Millennium Ecosystem Assessment, 2005. Ecosystems and human well-being: wetlands and water. World resources institute.

[CR30] Norsys, 2005. NETICA. Available at http://www.norsys.com).

[CR31] Pennington M, Gomes M, Donaldson C (2017). Handling Protest Responses in Contingent Valuation Surveys. Med Decis Making.

[CR32] Pollino CA, Woodberry O, Nicholson A, Korb K, Hart BT (2007). Parameterisation and evaluation of a Bayesian network for use in an ecological risk assessment. Environ Model Softw.

[CR33] R Core Team, 2020. R: A languange and snvironment for statistical computing. R Found. Stat. Comput. Vienna Austria.

[CR34] Reynolds CS (2000). Hydroecology of river plankton: the role of variability in channel flow. Hydrol Process.

[CR35] Ripley, B, Venables, B, Bates, D, Hornik, K, Gebhardt, A, Firth, D, 2021. MASS - Modern Applied Statistics with S.

[CR36] Schultz PW, Gouveia VV, Cameron LD, Tankha G, Schmuck P, Franěk M (2005). Values and their Relationship to Environmental Concern and Conservation Behavior. J Cross-Cult Psychol.

[CR37] Stewart‐Koster B, Bunn SE, Mackay SJ, Poff NL, Naiman RJ, Lake PS (2010). The use of Bayesian networks to guide investments in flow and catchment restoration for impaired river ecosystems. Freshw Biol.

[CR38] Thiemer K, Schneider SC, Demars BOL (2021). Mechanical removal of macrophytes in freshwater ecosystems: Implications for ecosystem structure and function. Sci Total Environ.

[CR39] Vereecken H, Baetens J, Viaene P, Mostaert F, Meire P (2006). Ecological management of aquatic plants: effects in lowland streams. Hydrobiologia.

[CR40] Verhofstad MJJM, Bakker ES (2019). Classifying nuisance submerged vegetation depending on ecosystem services. Limnology.

[CR41] Villamagna AM, Murphy BR (2010). Ecological and socio-economic impacts of invasive water hyacinth (Eichhornia crassipes): a review. Freshw Biol.

[CR42] Wickham, H, Chang, W, Henry, L, Pedersen, TL, Takahashi, K, Wilke, C, Woo, K, Yutani, H, Dunnington, D, RStudio, 2020. ggplot2: Create Elegant Data Visualisations Using the Grammar of Graphics.

