# Supplementary material for: Drivers of Perceived Nuisance Growth by Aquatic Plants
Source: Environ Manage. 2023 Jan 11;71(5):1024–36. doi: 10.1007/s00267-022-01781-x (PMC9832253; doi:10.1007/s00267-022-01781-x)
Supplement: Supplementary file 1 — Supporting Information II [file 267_2022_1781_MOESM1_ESM.pdf]

Drivers for perceived nuisance growth of aquatic plants

Kirstine Thiemer[1,2*], Bart Immerzeel[2,**], Susanne Schneider[1,2], Keneilwe Sebola[3], Julie Coetzee[3], Mathieu Baldo[4], Gabrielle Thiebaut[4], Sabine Hilt[5], Jan Köhler[5], Sarah Faye Harpenslager[5,***], Jan E. Vermaat[2]

[1] Section for Nature based Solutions and Aquatic Ecology, Norwegian Institute for Water Research, Økernveien 94, 0579 Oslo, Norway.

[2] Faculty of Environmental Sciences and Natural Resource Management, Norwegian University of Life Sciences, P.O. Box 5003, 1430 Ås, Norway.

[3] Centre for Biological Control, Botany Department, Rhodes University, PO Box 94, Grahamstown 610, South Africa.

[4] UMR 6553 CNRS ECOBIO, Université de Rennes 1, 263 Avenue du Général, 35042 Rennes, France.

[5] Dept. of Community and Ecosystem Ecology, Leibniz Institute of Freshwater Ecology and Inland Fisheries, Müggelseedamm 301, 12587, Berlin, Germany.

[*]Corresponding author: kirstine.thiemer@niva.no. ORCID: 0000-0002-3287-9502

** Norwegian Institute for Nature Research, Sognsveien 68, 0855 Oslo.

*** B-Ware Research Centre, Postbus 6558, 6503 GB, Nijmegen, The Netherlands

Supplementary Information II

Description of a conceptual Bayesian network with probabilities obtained from the survey and with illustrative probabilities

The Bayesian network (BN) provides a first attempt to integrate societal and ecological perspectives of macrophyte mass developments and macrophyte removal. Water managers can manipulate the BN to explore and identify the best management options of macrophyte mass development to different user groups (swimmers, boater, anglers, residents/visitors) and at the same time consider the risk under different scenarios (Fig. 2 in main text). Water managers may also set the risk of phytoplankton bloom (endpoint) to a specific target and see how probabilities are affected backwards throughout the whole BN, identifying key nodes on which the set target depends. In this BN, the state probability of the node *Perception* (Table S1) were conditioned from the parent nodes *Macrophyte species, Macrophyte growth, Activity* and *Respondent type*. The probabilities of nuisance (1- probability for nuisance = no nuisance) were calculated for all combinations by using data collected in a comprehensive survey study and generalized linear mixed models (GLMMs). *Macrophyte removal* (Table S2) were conditioned by the *Perception* parent node. These probabilities could not be quantified directly from the surveys, but the instead a simple inverse relationship between perception and macrophyte removal that also depends on macrophyte growth, was assumed (Table S3). Lastly, the link between *Macrophyte removal* and the network to *Phytoplankton* development is obtained from the BN developed by Thiemer et al. (2021). This part of the BN describes one short-term consequences of removing aquatic plants that is increased risk of phytoplankton bloom (Kuiper et al. 2017) (Fig. S1). Phytoplankton development dependent on (i) the type of *ecosystems* (flowing or standing water with submerged plants, standing water with floating plants and the degree of *plant removal*, and (ii) on the balance between *resources* (growth potential) and *disturbances* (loss processes) – Reynolds (1984). The state probabilities of *phytoplankton* (Table S4) development were conditioned symmetrically from the parent nodes *resources* and *disturbances* to recognise their equivalent strength for equivalent set of states. For example, high resources and low disturbances will very likely (100%) produce a phytoplankton bloom, while low resources and moderate disturbances will likely result in low (75%) to moderate (25%) phytoplankton abundance. *Resources* (growth potential, Table S5) may include *light* and *nutrient loading* (C, N, P) and *benthic fish foraging*. The latter can resuspend the sediment, increase nutrient supply and phytoplankton growth (Matsuzaki *et al.*, 2009), assuming it does not substantially affect light availability. *Disturbances* (removal of biomass, Table S6) may be characterised by predation (*zooplankton*) and turbulence and water retention time (*flow*) conditions in rivers and lakes (Bernes *et al.*, 2015; Reynolds, 2000; Gallardo *et al.*, 2009). *Plant removal* and *ecosystem* types will affect *flow* (turbulence, water renewal; Table S7) and *light* (Table S8) directly. Aquatic plant removal in all freshwater ecosystems will affect *benthic fish foraging* and *zooplankton* through changes in benthic and pelagic trophic interactions. Aquatic plant architecture affects macroinvertebrate richness, abundance and functional feeding groups (e.g. Taniguchi, Nakano & Tokeshi, 2003; Demars *et al.*, 2012; Hansen *et al.*, 2011). When aquatic plants are removed so are the associated epiphytes and likely most of the grazers, thereby depriving fish (Jones & Sayer, 2003; Bécares *et al.*, 2008). Fish can rapidly change their mode of foraging when prey density suddenly decline (e.g. Fausch, Nakano & Kitano, 1997) and may shift to *benthic foraging* (Table S9) when supply of *epiphytic invertebrates* (Table S10) declines in response to total removal of macrophytes or in ecosystems dominated by floating plants (Kornijów, Measey & Moss, 2016; Carpenter, van Donk & Wetzel, 1998). Phytoplankton blooms are also dependent on the trophic cascade *piscivorous fish* (Table S11) > *planktivorous fish* (Table S12) > *zooplankton* (Table S13) > *phytoplankton*, as seen through whole lake biomanipulations (Bernes *et al.*, 2015). Conditional probability tables used in the BN (Table S4 to S13)

were derived from general knowledge in ecology (*op. cit.*), but remain hypothetical and should only be used for illustrative purpose.

Table S1. Conditional probability table (in %) for perceiving macrophyte growth as a nuisance abundance with respect to macrophyte species, respondent type, activity use, macrophyte growth level. Probabilities arrives from the GLLMs. *x* indicates that probabilities were not relevant for this combination.

				Perception	
**Species**	**Respondent**	**Activity**	**Macrophyte level**	**Nuisance**	**No nuisance**
E. nuttallii	Resident	Swimming	1	x	x
E. nuttallii	Resident	Swimming	2	x	x
E. nuttallii	Resident	Swimming	3	x	x
E. nuttallii	Resident	Swimming	4	x	x
E. nuttallii	Resident	Swimming	5	x	x
E. nuttallii	Resident	Boating	1	11.1	88.9
E. nuttallii	Resident	Boating	2	26.5	73.5
E. nuttallii	Resident	Boating	3	50.9	49.1
E. nuttallii	Resident	Boating	4	74.9	25.1
E. nuttallii	Resident	Boating	5	89.6	10.4
E. nuttallii	Resident	Angling	1	2.9	97.1
E. nuttallii	Resident	Angling	2	7.9	92.1
E. nuttallii	Resident	Angling	3	19.8	80.2
E. nuttallii	Resident	Angling	4	41.5	58.5
E. nuttallii	Resident	Angling	5	67.2	32.8
E. nuttallii	Resident	Appreciation of biodiversity	1	3.5	96.5
E. nuttallii	Resident	Appreciation of biodiversity	2	9.5	90.5
E. nuttallii	Resident	Appreciation of biodiversity	3	23.2	76.8
E. nuttallii	Resident	Appreciation of biodiversity	4	46.5	53.5
E. nuttallii	Resident	Appreciation of biodiversity	5	71.5	28.5
E. nuttallii	Resident	Appreciation of landscape	1	4.5	95.5
E. nuttallii	Resident	Appreciation of landscape	2	11.9	88.1
E. nuttallii	Resident	Appreciation of landscape	3	28.1	71.9
E. nuttallii	Resident	Appreciation of landscape	4	53	47
E. nuttallii	Resident	Appreciation of landscape	5	76.5	23.5
E. nuttallii	Resident	Birdwatching	1	x	x
E. nuttallii	Resident	Birdwatching	2	x	x
E. nuttallii	Resident	Birdwatching	3	x	x
E. nuttallii	Resident	Birdwatching	4	x	x
E. nuttallii	Resident	Birdwatching	5	x	x
E. nuttallii	Visitor	Swimming	1	x	x
E. nuttallii	Visitor	Swimming	2	x	x
E. nuttallii	Visitor	Swimming	3	x	x
E. nuttallii	Visitor	Swimming	4	x	x
E. nuttallii	Visitor	Swimming	5	x	x
E. nuttallii	Visitor	Boating	1	6.2	93.8

Species	Respondent	Activity	Macrophyte level	Perception	
				Nuisance	No nuisance
E. nuttallii	Visitor	Boating	2	15.9	84.1
E. nuttallii	Visitor	Boating	3	35.3	64.7
E. nuttallii	Visitor	Boating	4	61.1	38.9
E. nuttallii	Visitor	Boating	5	81.9	18.1
E. nuttallii	Visitor	Angling	1	1.5	98.5
E. nuttallii	Visitor	Angling	2	4.3	95.7
E. nuttallii	Visitor	Angling	3	11.4	88.6
E. nuttallii	Visitor	Angling	4	27.1	72.9
E. nuttallii	Visitor	Angling	5	51.8	48.2
E. nuttallii	Visitor	Appreciation of biodiversity	1	1.9	98.1
E. nuttallii	Visitor	Appreciation of biodiversity	2	5.2	94.8
E. nuttallii	Visitor	Appreciation of biodiversity	3	13.7	86.3
E. nuttallii	Visitor	Appreciation of biodiversity	4	31.3	68.7
E. nuttallii	Visitor	Appreciation of biodiversity	5	56.8	43.2
E. nuttallii	Visitor	Appreciation of landscape	1	2.4	97.6
E. nuttallii	Visitor	Appreciation of landscape	2	6.6	93.4
E. nuttallii	Visitor	Appreciation of landscape	3	17	83
E. nuttallii	Visitor	Appreciation of landscape	4	37.2	62.8
E. nuttallii	Visitor	Appreciation of landscape	5	63	37
E. nuttallii	Visitor	Birdwatching	1	x	x
E. nuttallii	Visitor	Birdwatching	2	x	x
E. nuttallii	Visitor	Birdwatching	3	x	x
E. nuttallii	Visitor	Birdwatching	4	x	x
E. nuttallii	Visitor	Birdwatching	5	x	x
P. crassipes	Resident	Swimming	1	18.4	81.6
P. crassipes	Resident	Swimming	2	31.1	68.9
P. crassipes	Resident	Swimming	3	47.5	52.5
P. crassipes	Resident	Swimming	4	64.4	35.6
P. crassipes	Resident	Swimming	5	78.4	21.6
P. crassipes	Resident	Boating	1	19.1	80.9
P. crassipes	Resident	Boating	2	32.2	67.8
P. crassipes	Resident	Boating	3	48.8	51.2
P. crassipes	Resident	Boating	4	65.5	34.5
P. crassipes	Resident	Boating	5	79.2	20.8
P. crassipes	Resident	Angling	1	17.9	82.1
P. crassipes	Resident	Angling	2	30.4	69.6
P. crassipes	Resident	Angling	3	46.7	53.3
P. crassipes	Resident	Angling	4	63.7	36.3
P. crassipes	Resident	Angling	5	77.8	22.2
P. crassipes	Resident	Appreciation of biodiversity	1	17.8	82.2
P. crassipes	Resident	Appreciation of biodiversity	2	30.2	69.8
P. crassipes	Resident	Appreciation of biodiversity	3	46.6	53.4
P. crassipes	Resident	Appreciation of biodiversity	4	63.4	36.6
P. crassipes	Resident	Appreciation of biodiversity	5	77.7	22.3

Species	Respondent	Activity	Macrophyte level	Perception	
				Nuisance	No nuisance
P. crassipes	Resident	Appreciation of landscape	1	19.9	80.1
P. crassipes	Resident	Appreciation of landscape	2	33.3	66.7
P. crassipes	Resident	Appreciation of landscape	3	50	50
P. crassipes	Resident	Appreciation of landscape	4	66.7	33.3
P. crassipes	Resident	Appreciation of landscape	5	80	20
P. crassipes	Resident	Birdwatching	1	x	x
P. crassipes	Resident	Birdwatching	2	x	x
P. crassipes	Resident	Birdwatching	3	x	x
P. crassipes	Resident	Birdwatching	4	x	x
P. crassipes	Resident	Birdwatching	5	x	x
P. crassipes	Visitor	Swimming	1	23.3	76.7
P. crassipes	Visitor	Swimming	2	31.1	68.9
P. crassipes	Visitor	Swimming	3	10	90
P. crassipes	Visitor	Swimming	4	49.7	50.3
P. crassipes	Visitor	Swimming	5	59.4	40.6
P. crassipes	Visitor	Boating	1	38.3	61.7
P. crassipes	Visitor	Boating	2	47.9	52.1
P. crassipes	Visitor	Boating	3	57.6	42.4
P. crassipes	Visitor	Boating	4	66.8	33.2
P. crassipes	Visitor	Boating	5	74.8	25.2
P. crassipes	Visitor	Angling	1	36.1	63.9
P. crassipes	Visitor	Angling	2	45.6	54.4
P. crassipes	Visitor	Angling	3	55.3	44.7
P. crassipes	Visitor	Angling	4	64.7	35.3
P. crassipes	Visitor	Angling	5	73	27
P. crassipes	Visitor	Appreciation of biodiversity	1	35.8	64.2
P. crassipes	Visitor	Appreciation of biodiversity	2	45.2	54.8
P. crassipes	Visitor	Appreciation of biodiversity	3	54.9	45.1
P. crassipes	Visitor	Appreciation of biodiversity	4	64.3	35.7
P. crassipes	Visitor	Appreciation of biodiversity	5	72.8	27.2
P. crassipes	Visitor	Appreciation of landscape	1	28.1	71.9
P. crassipes	Visitor	Appreciation of landscape	2	36.6	63.4
P. crassipes	Visitor	Appreciation of landscape	3	46.1	53.9
P. crassipes	Visitor	Appreciation of landscape	4	55.9	44.1
P. crassipes	Visitor	Appreciation of landscape	5	65.2	34.8
P. crassipes	Visitor	Birdwatching	1	x	x
P. crassipes	Visitor	Birdwatching	2	x	x
P. crassipes	Visitor	Birdwatching	3	x	x
P. crassipes	Visitor	Birdwatching	4	x	x
P. crassipes	Visitor	Birdwatching	5	x	x
Ludwigia spp.	Resident	Swimming	1	x	x
Ludwigia spp.	Resident	Swimming	2	x	x
Ludwigia spp.	Resident	Swimming	3	x	x
Ludwigia spp.	Resident	Swimming	4	x	x

Species	Respondent	Activity	Macrophyte level	Perception	
				Nuisance	No nuisance
Ludwigia spp.	Resident	Swimming	5	x	x
Ludwigia spp.	Resident	Boating	1	17.8	82.2
Ludwigia spp.	Resident	Boating	2	16.2	83.8
Ludwigia spp.	Resident	Boating	3	15.2	84.8
Ludwigia spp.	Resident	Boating	4	13.9	86.1
Ludwigia spp.	Resident	Boating	5	12.8	87.2
Ludwigia spp.	Resident	Angling	1	17.6	82.4
Ludwigia spp.	Resident	Angling	2	16.3	83.7
Ludwigia spp.	Resident	Angling	3	15	85
Ludwigia spp.	Resident	Angling	4	13.8	86.2
Ludwigia spp.	Resident	Angling	5	12.7	87.3
Ludwigia spp.	Resident	Appreciation of biodiversity	1	19.3	80.7
Ludwigia spp.	Resident	Appreciation of biodiversity	2	17.8	82.2
Ludwigia spp.	Resident	Appreciation of biodiversity	3	16.5	83.5
Ludwigia spp.	Resident	Appreciation of biodiversity	4	15.2	84.8
Ludwigia spp.	Resident	Appreciation of biodiversity	5	13.9	86.1
Ludwigia spp.	Resident	Appreciation of landscape	1	17.2	82.8
Ludwigia spp.	Resident	Appreciation of landscape	2	15.9	84.1
Ludwigia spp.	Resident	Appreciation of landscape	3	14.6	85.4
Ludwigia spp.	Resident	Appreciation of landscape	4	13.5	86.5
Ludwigia spp.	Resident	Appreciation of landscape	5	12.4	87.6
Ludwigia spp.	Resident	Birdwatching	1	18.4	81.6
Ludwigia spp.	Resident	Birdwatching	2	16.9	83.1
Ludwigia spp.	Resident	Birdwatching	3	15.7	84.3
Ludwigia spp.	Resident	Birdwatching	4	14.4	85.6
Ludwigia spp.	Resident	Birdwatching	5	13.3	86.7
Ludwigia spp.	Visitor	Swimming	1	x	x
Ludwigia spp.	Visitor	Swimming	2	x	x
Ludwigia spp.	Visitor	Swimming	3	x	x
Ludwigia spp.	Visitor	Swimming	4	x	x
Ludwigia spp.	Visitor	Swimming	5	x	x
Ludwigia spp.	Visitor	Boating	1	15.6	84.4
Ludwigia spp.	Visitor	Boating	2	16	84
Ludwigia spp.	Visitor	Boating	3	16.4	83.6
Ludwigia spp.	Visitor	Boating	4	16.8	83.2
Ludwigia spp.	Visitor	Boating	5	17.1	82.9
Ludwigia spp.	Visitor	Angling	1	15.5	84.5
Ludwigia spp.	Visitor	Angling	2	15.9	84.1
Ludwigia spp.	Visitor	Angling	3	16.2	83.8
Ludwigia spp.	Visitor	Angling	4	16.6	83.4
Ludwigia spp.	Visitor	Angling	5	17	83
Ludwigia spp.	Visitor	Appreciation of biodiversity	1	16.9	83.1
Ludwigia spp.	Visitor	Appreciation of biodiversity	2	17.4	82.6
Ludwigia spp.	Visitor	Appreciation of biodiversity	3	17.8	82.2

Species	Respondent	Activity	Macrophyte level	Perception	
				Nuisance	No nuisance
Ludwigia spp.	Visitor	Appreciation of biodiversity	4	18.2	81.8
Ludwigia spp.	Visitor	Appreciation of biodiversity	5	18.6	81.4
Ludwigia spp.	Visitor	Appreciation of landscape	1	15.1	84.9
Ludwigia spp.	Visitor	Appreciation of landscape	2	15.5	84.5
Ludwigia spp.	Visitor	Appreciation of landscape	3	15.8	84.2
Ludwigia spp.	Visitor	Appreciation of landscape	4	16.2	83.8
Ludwigia spp.	Visitor	Appreciation of landscape	5	16.6	83.4
Ludwigia spp.	Visitor	Birdwatching	1	16.2	83.8
Ludwigia spp.	Visitor	Birdwatching	2	16.5	83.5
Ludwigia spp.	Visitor	Birdwatching	3	16.9	83.1
Ludwigia spp.	Visitor	Birdwatching	4	17.3	82.7
Ludwigia spp.	Visitor	Birdwatching	5	17.7	82.3
J. bulbosus	Resident	Swimming	1	29	71
J. bulbosus	Resident	Swimming	2	43.3	56.7
J. bulbosus	Resident	Swimming	3	59.1	40.9
J. bulbosus	Resident	Swimming	4	73.2	26.8
J. bulbosus	Resident	Swimming	5	83.8	16.2
J. bulbosus	Resident	Boating	1	27.8	72.2
J. bulbosus	Resident	Boating	2	42.1	57.9
J. bulbosus	Resident	Boating	3	57.9	42.1
J. bulbosus	Resident	Boating	4	72.2	27.8
J. bulbosus	Resident	Boating	5	83.1	16.9
J. bulbosus	Resident	Angling	1	25.8	74.2
J. bulbosus	Resident	Angling	2	39.7	60.3
J. bulbosus	Resident	Angling	3	55.4	44.6
J. bulbosus	Resident	Angling	4	70.2	29.8
J. bulbosus	Resident	Angling	5	81.7	18.3
J. bulbosus	Resident	Appreciation of biodiversity	1	22.7	77.3
J. bulbosus	Resident	Appreciation of biodiversity	2	35.7	64.3
J. bulbosus	Resident	Appreciation of biodiversity	3	51.2	48.8
J. bulbosus	Resident	Appreciation of biodiversity	4	66.5	33.5
J. bulbosus	Resident	Appreciation of biodiversity	5	79	21
J. bulbosus	Resident	Appreciation of landscape	1	25.7	74.3
J. bulbosus	Resident	Appreciation of landscape	2	39.5	60.5
J. bulbosus	Resident	Appreciation of landscape	3	55.3	44.7
J. bulbosus	Resident	Appreciation of landscape	4	70	30
J. bulbosus	Resident	Appreciation of landscape	5	81.6	18.4
J. bulbosus	Resident	Birdwatching	1	x	x
J. bulbosus	Resident	Birdwatching	2	x	x
J. bulbosus	Resident	Birdwatching	3	x	x
J. bulbosus	Resident	Birdwatching	4	x	x
J. bulbosus	Resident	Birdwatching	5	x	x
J. bulbosus	Visitor	Swimming	1	12.2	87.8
J. bulbosus	Visitor	Swimming	2	20.8	79.2

Species	Respondent	Activity	Macrophyte level	Perception	
				Nuisance	No nuisance
J. bulbosus	Visitor	Swimming	3	33.3	66.7
J. bulbosus	Visitor	Swimming	4	48.5	51.5
J. bulbosus	Visitor	Swimming	5	64.1	35.9
J. bulbosus	Visitor	Boating	1	11.7	88.3
J. bulbosus	Visitor	Boating	2	20.1	79.9
J. bulbosus	Visitor	Boating	3	32.2	67.8
J. bulbosus	Visitor	Boating	4	47.4	52.6
J. bulbosus	Visitor	Boating	5	62.9	37.1
J. bulbosus	Visitor	Angling	1	10.7	89.3
J. bulbosus	Visitor	Angling	2	18.5	81.5
J. bulbosus	Visitor	Angling	3	30	70
J. bulbosus	Visitor	Angling	4	44.8	55.2
J. bulbosus	Visitor	Angling	5	60.6	39.4
J. bulbosus	Visitor	Appreciation of biodiversity	1	9.2	90.8
J. bulbosus	Visitor	Appreciation of biodiversity	2	16.1	83.9
J. bulbosus	Visitor	Appreciation of biodiversity	3	26.6	73.4
J. bulbosus	Visitor	Appreciation of biodiversity	4	40.7	59.3
J. bulbosus	Visitor	Appreciation of biodiversity	5	56.5	43.5
J. bulbosus	Visitor	Appreciation of landscape	1	10.6	89.4
J. bulbosus	Visitor	Appreciation of landscape	2	18.4	81.6
J. bulbosus	Visitor	Appreciation of landscape	3	29.9	70.1
J. bulbosus	Visitor	Appreciation of landscape	4	44.7	55.3
J. bulbosus	Visitor	Appreciation of landscape	5	60.4	39.6
J. bulbosus	Visitor	Birdwatching	1	x	x
J. bulbosus	Visitor	Birdwatching	2	x	x
J. bulbosus	Visitor	Birdwatching	3	x	x
J. bulbosus	Visitor	Birdwatching	4	x	x
J. bulbosus	Visitor	Birdwatching	5	x	x
S. sagittifolia	Resident	Swimming	1	2.1	97.9
S. sagittifolia	Resident	Swimming	2	7.3	92.7
S. sagittifolia	Resident	Swimming	3	22.6	77.4
S. sagittifolia	Resident	Swimming	4	52	48
S. sagittifolia	Resident	Swimming	5	80	20
S. sagittifolia	Resident	Boating	1	2.1	97.9
S. sagittifolia	Resident	Boating	2	7.8	92.2
S. sagittifolia	Resident	Boating	3	23.6	76.4
S. sagittifolia	Resident	Boating	4	53.4	46.6
S. sagittifolia	Resident	Boating	5	80.1	19.9
S. sagittifolia	Resident	Angling	1	2.6	97.4
S. sagittifolia	Resident	Angling	2	8.9	91.1
S. sagittifolia	Resident	Angling	3	26.7	73.3
S. sagittifolia	Resident	Angling	4	57.4	42.6
S. sagittifolia	Resident	Angling	5	83.3	16.7
S. sagittifolia	Resident	Appreciation of biodiversity	1	1.5	98.5

Species	Respondent	Activity	Macrophyte level	Perception	
				Nuisance	No nuisance
S. sagittifolia	Resident	Appreciation of biodiversity	2	3.8	96.2
S. sagittifolia	Resident	Appreciation of biodiversity	3	12.7	87.3
S. sagittifolia	Resident	Appreciation of biodiversity	4	35	65
S. sagittifolia	Resident	Appreciation of biodiversity	5	66.6	33.4
S. sagittifolia	Resident	Appreciation of landscape	1	1.9	98.1
S. sagittifolia	Resident	Appreciation of landscape	2	6.9	93.1
S. sagittifolia	Resident	Appreciation of landscape	3	21.6	78.4
S. sagittifolia	Resident	Appreciation of landscape	4	50.5	49.5
S. sagittifolia	Resident	Appreciation of landscape	5	79.1	20.9
S. sagittifolia	Resident	Birdwatching	1	x	x
S. sagittifolia	Resident	Birdwatching	2	x	x
S. sagittifolia	Resident	Birdwatching	3	x	x
S. sagittifolia	Resident	Birdwatching	4	x	x
S. sagittifolia	Resident	Birdwatching	5	x	x
S. sagittifolia	Visitor	Swimming	1	5.1	94.9
S. sagittifolia	Visitor	Swimming	2	10.7	89.3
S. sagittifolia	Visitor	Swimming	3	21.1	78.9
S. sagittifolia	Visitor	Swimming	4	37.3	62.7
S. sagittifolia	Visitor	Swimming	5	56.9	43.1
S. sagittifolia	Visitor	Boating	1	5.4	94.6
S. sagittifolia	Visitor	Boating	2	11.2	88.8
S. sagittifolia	Visitor	Boating	3	21.9	78.1
S. sagittifolia	Visitor	Boating	4	38.5	61.5
S. sagittifolia	Visitor	Boating	5	58.3	41.7
S. sagittifolia	Visitor	Angling	1	6.3	93.7
S. sagittifolia	Visitor	Angling	2	13	87
S. sagittifolia	Visitor	Angling	3	24.9	75.1
S. sagittifolia	Visitor	Angling	4	42.5	57.5
S. sagittifolia	Visitor	Angling	5	62.2	37.8
S. sagittifolia	Visitor	Appreciation of biodiversity	1	2.6	97.4
S. sagittifolia	Visitor	Appreciation of biodiversity	2	56.3	43.7
S. sagittifolia	Visitor	Appreciation of biodiversity	3	11.7	88.3
S. sagittifolia	Visitor	Appreciation of biodiversity	4	22.8	77.2
S. sagittifolia	Visitor	Appreciation of biodiversity	5	39.7	60.3
S. sagittifolia	Visitor	Appreciation of landscape	1	4.8	95.2
S. sagittifolia	Visitor	Appreciation of landscape	2	10.2	89.8
S. sagittifolia	Visitor	Appreciation of landscape	3	20.1	79.9
S. sagittifolia	Visitor	Appreciation of landscape	4	35.9	64.1
S. sagittifolia	Visitor	Appreciation of landscape	5	55.5	44.5
S. sagittifolia	Visitor	Birdwatching	1	x	x
S. sagittifolia	Visitor	Birdwatching	2	x	x
S. sagittifolia	Visitor	Birdwatching	3	x	x
S. sagittifolia	Visitor	Birdwatching	4	x	x
S. sagittifolia	Visitor	Birdwatching	5	x	x

Table S2. Conditional probability table (in %) for macrophyte removal with respect to perception. Rational: invese relationship. Based on Table S3.

	Macrophyte removal		
**Perception**	**Non**	**Partial**	**Full**
Nuisance	20	20	60
No Nuisance	80	20	0

Table. S3. Background for making the probability tables when linking perception with removal practice. Rationale: simple inverse relationship for nuisance and no nuisance. Green – No removal, Yellow – partial removal and Orange – full removal of macrophytes. Example of calculating the probabilities for Nuisance (number of cells/total number of cells (15)): Full removal: 9/15 = 0.60, partial: 3/15 = 0.20 and no removal: 3/15 =0.20 (Table S2).

**Macrophyte level**		**No nuisance**			**Nuisance**	
**1**	0	0	0	0	0	1
**2**	0	0	0	0	1	1
**3**	0	0	0	1	1	1
**4**	0	0	1	1	1	1
**5**	0	1	1	1	1	1

Table S4. Conditional probability table (in %) for phytoplankton abundance with respect to disturbance and resources. Rational: resources and disturbances could play an equal role, as reflected in the symmetry of the conditional probabilities.

		Phytoplankton		
**Resources**	**Disturbances**	**Low**	**Moderate**	**High**
Low	Low	50	50	0
Low	Moderate	75	25	0
Low	High	100	0	0
Moderate	Low	0	50	50
Moderate	Moderate	0	100	0
Moderate	High	50	50	0
High	Low	0	0	100
High	Moderate	0	50	50
High	High	0	100	0

Table S5. Conditional probability table (in %) for resources with respect to light, nutrient loading and benthic fish foraging. Rational: Low light only allows a maximum of 50% moderate resources whatever nutrient loading and benthic fish foraging. High light allows for maximum exploitation of other resources and 100% high resources. Under high light, nutrient loading plays a major role, with fish benthic foraging playing a subordinate additional role, 25-75% moderate resource under low nutrient loading, decreasing to 25-45% high resource under moderate nutrient loading, and playing no additional role under high nutrient loading.

			Resources		
**Light**	**Nutrient loading**	**Benthic fish foraging**	**Low**	**Moderate**	**High**
Low	Low	Low	100	0	0
Low	Low	Moderate	90	10	0
Low	Low	High	80	20	0
Low	Moderate	Low	75	25	0
Low	Moderate	Moderate	70	30	0
Low	Moderate	High	65	35	0
Low	High	Low	50	50	0
Low	High	Moderate	50	50	0
Low	High	High	50	50	0
High	Low	Low	75	25	0
High	Low	Moderate	50	50	0
High	Low	High	25	75	0
High	Moderate	Low	0	75	25
High	Moderate	Moderate	0	65	35
High	Moderate	High	0	55	45
High	High	Low	0	0	100
High	High	Moderate	0	0	100
High	High	High	0	0	100

Table S6. Conditional probability table (in %) for disturbance with respect to flow (i.e. turbulence and water renewal) and zooplankton abundance. Rational: Flow was set as a primary control, with biomass removal exceeding zooplankton growth. Thus, high zooplankton was not possible under moderate flow, and similarly zooplankton could only be in low abundance under high flow. Impossible combinations of states were indicated with crosses (x).

		Disturbance		
**Flow**	**Zooplankton**	**Low**	**Moderate**	**High**
Low	Low	100	0	0
Low	Moderate	0	100	0
Low	High	0	75	25
Moderate	Low	0	100	0
Moderate	Moderate	0	50	50
Moderate	High	x	x	x
High	Low	0	0	100
High	Moderate	x	x	x
High	High	x	x	x

Table S7. Conditional probability table (in %) for flow with respect to plant removal and ecosystem. Rational: Standing submerged may also represent ponded sections of river under drought conditions. Low water retention time (or high water renewal) in flowing submerged ecosystems does not allow plankton to develop, except in some small pockets, e.g. large rivers, connected backwaters and impounded sections (Reynolds, 2000; Neal *et al.*, 2006; Soballe & Kimmel, 1987). The effects of partial and full removal of submerged plants were partly derived from experimental results summarised in Appendix 1, section hydraulics / rivers.

		Flow		
**Plant removal**	**Ecosystem**	**Low**	**Medium**	**High**
No	Standing floating	100	0	0
No	Standing submerged	100	0	0
No	Flowing submerged	75	25	0
Partial	Standing floating	100	0	0
Partial	Standing submerged	100	0	0
Partial	Flowing submerged	0	50	50
Full	Standing floating	100	0	0
Full	Standing submerged	100	0	0
Full	Flowing submerged	0	0	100

Table S8. Conditional probability table (in %) for light (in the water column) with respect to plant removal and ecosystem type. Rational: High *light* represents favourable light conditions for phytoplankton growth when macrophyte cover is low.

		Light	
**Plant removal**	**Ecosystem**	**Low**	**High**
No	Standing floating	100	0
No	Standing submerged	50	50
No	Flowing submerged	50	50
Partial	Standing floating	50	50
Partial	Standing submerged	25	75
Partial	Flowing submerged	25	75
Full	Standing floating	0	100
Full	Standing submerged	0	100
Full	Flowing submerged	0	100

Table S9. Conditional probability table (in %) for benthic fish foraging with respect to epiphytic invertebrates. Rational: a simple inverse relationship was assumed, together with feeding plasticity in fish species present.

	Benthic fish foraging		
**Epiphytic invertebrates**	**Low**	**Moderate**	**High**
Low	0	0	100
Moderate	0	100	0
High	100	0	0

Table S10. Conditional probability table (in %) for epiphytic invertebrates with respect to plant removal and ecosystem.

		Epiphytic invertebrates		
**Plant removal**	**Ecosystem**	**Low**	**Moderate**	**High**
No	Standing floating	75	25	0
No	Standing submerged	0	25	75
No	Flowing submerged	0	25	75
Partial	Standing floating	50	50	0
Partial	Standing submerged	25	75	0
Partial	Flowing submerged	25	75	0
Full	Standing floating	25	50	25
Full	Standing submerged	100	0	0
Full	Flowing submerged	100	0	0

Table S11. Conditional probability table (in %) for piscivorous fish predation with respect to plant removal and piscivorous fish. Rational: without plant removal, fish predation should be low because piscivorous fish cannot hunt efficiently within dense macrophyte beds. With a partial removal, fish production may be optimised through the provision of refugia for planktivorous fish, and space for piscivorous fish to hide and hunt at edge of patches. With full plant removal, predator avoidance by planktivorous fish is severely impaired and piscivorous fish predation should be high (at least in the short term).

		Piscivorous fish predation	
**Plant removal**	**Piscivorous fish**	**Low**	**High**
No	Absent	100	0
No	Present	100	0
Partial	Absent	100	0
Partial	Present	50	50
Full	Absent	100	0
Full	Present	0	100

Table S12. Conditional probability table (in %) for planktivorous fish with respect to piscivorous fish predation. Rational: The trophic cascade effect of piscivorous fish has been well documented (see Fig. 13, 14 in Bernes *et al.*, 2015).

	Planktivorous fish	
**Piscivorous fish predation**	**Low**	**High**
Low	0	100
Moderate	50	50
High	100	0

Table S13. Conditional probability table (in %) for zooplankton with respect to flow and planktivorous fish. Rational: Zooplankton abundance will be primarily constrained by turbulence and water renewal (flow), with additional pressure through predation by planktivorous fish (Bernes *et al.*, 2015). High flow exceeds the growth potential of zooplankton, and thus zooplankton abundance remains low.

		Zooplankton		
**Flow**	**Planktivorous fish**	**Low**	**Moderate**	**High**
Low	Low	0	0	100
Low	High	50	50	0
Moderate	Low	0	100	0
Moderate	High	75	25	0
High	Low	100	0	0
High	High	100	0	0

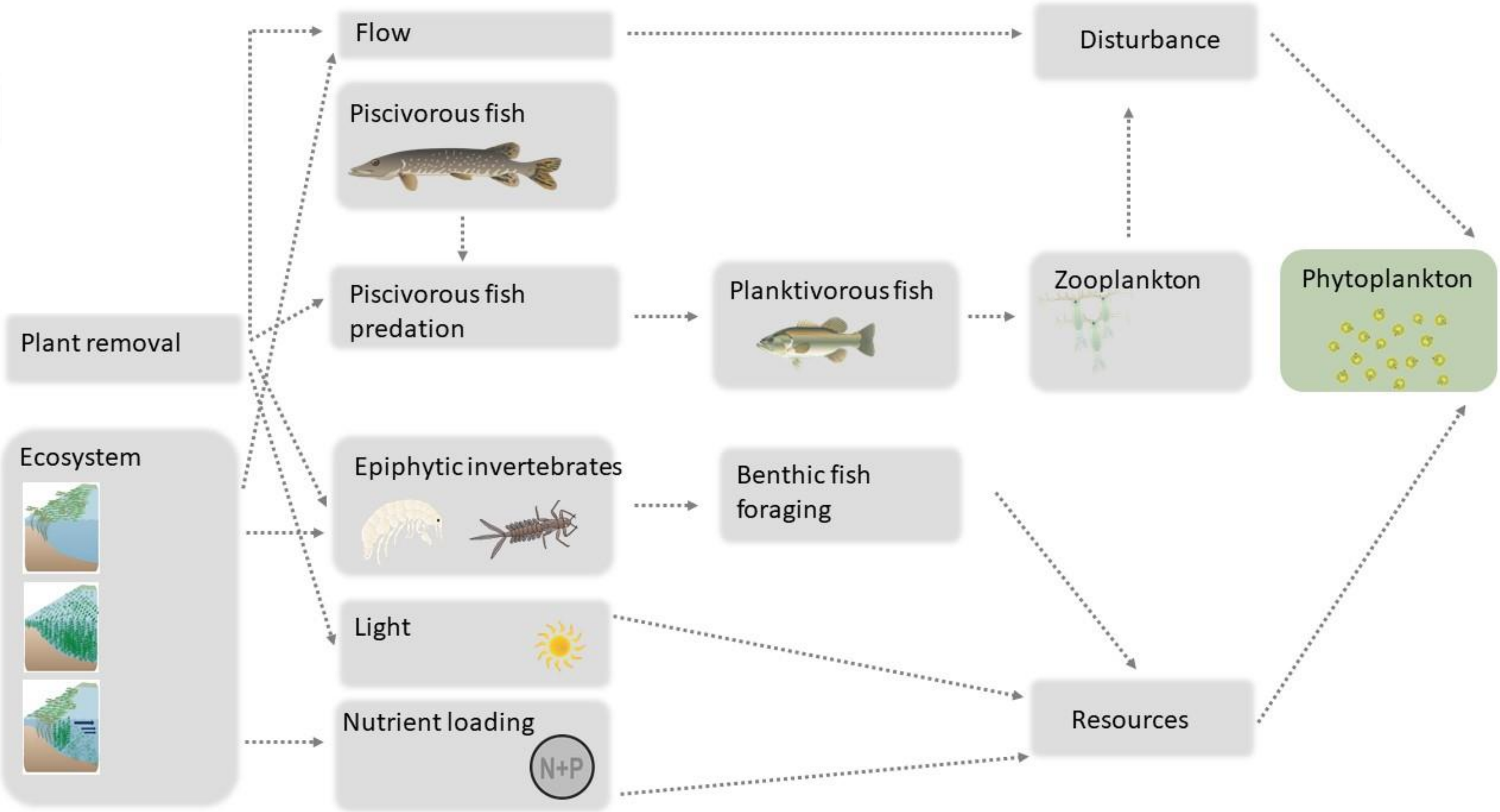

Figure S1. Conceptual diagram of key determinants influencing phytoplankton abundance. Symbols in the figure are from the Integration and Application Network, Univ. of Maryland Center for Environmental Science (ian.umces.edu/symbols/).

References

Bécares E., Goma J., Fernandez-Alaez M., Fernandez-Alaez C., Romo S., Miracle M.R., Stahl-Delbanco A., Hansson L.-A., Gyllstrom M., Van De Bund W.J., Van Donk E., Kairesalo T., Hietala J., Stephen D., Balayla D. & Moss B. (2008) Effects of nutrients and fish on periphyton and plant biomass across a European latitudinal gradient. *Aquatic Ecology,* **42,** 561-574.

Bernes C., Carpenter S.R., Gårdmark A., Larsson P., Persson L., Skov C., Speed J.D. & Van Donk E. (2015) What is the influence of a reduction of planktivorous and benthivorous fish on water quality in temperate eutrophic lakes? A systematic review. *Environmental Evidence,* **4,** 1-28.

Bronmark C. (1994) Effects of tench and perch on interactions in a freshwater, benthic food chain. *Ecology,* **75,** 1818-1828.

Carpenter S.R., Van Donk E. & Wetzel R.G. (1998) Nutrient-loading gradient in shallow lakes: report of the group discussion. In: *The structuring role of submerged macrophytes in lakes.* (Eds E. Jeppesen & M. Søndergaard & M. Søndergaard & K. Christoffersen), pp. 393-396. Springer Verlag, New York.

Demars B.O.L., Kemp J.L., Friberg N., Usseglio-Polatera P. & Harper D.M. (2012) Linking biotopes to invertebrates in rivers: biological traits, taxonomic composition and diversity. *Ecological Indicators,* **23,** 301-311.

Fausch K.D., Nakano S. & Kitano S. (1997) Experimentally induced foraging mode shift by sympatric charrs in a Japanese mountain stream. *Behavioral Ecology,* **8,** 414-420.

Gallardo B., Gascon S., Gonzalez-Sanchis M., Cabezas A. & Comin F.A. (2009) Modelling the response of floodplain aquatic assemblages across the lateral hydrological connectivity gradient. *Marine and Freshwater Research,* **60,** 924-935.

Genkai-Kato M. (2007) Macrophyte refuges, prey behaviour and trophic interactions: consequences for lake water clarity. *Ecology Letters,* **10,** 105-114.

Graham S.E., O'brien J.M., Burrell T.K. & Mcintosh A.R. (2015) Aquatic macrophytes alter productivity-richness relationships in eutrophic stream food webs. *Ecosphere,* **6**.

Gross E.M. (2003) Allelopathy of aquatic autotrophs. *Critical Reviews in Plant Sciences,* **22,** 313-339.

Hansen J.P., Wikstrom S.A., Axemar H. & Kautsky L. (2011) Distribution differences and active habitat choices of invertebrates between macrophytes of different morphological complexity. *Aquatic Ecology,* **45,** 11-22.

Harrison S.S.C., Bradley D.C. & Harris I.T. (2005) Uncoupling strong predator-prey interactions in streams: the role of marginal macrophytes. *Oikos,* **108,** 433-448.

Hu F.J., Bolding K., Bruggeman J., Jeppesen E., Flindt M.R., Van Gerven L., Janse J.H., Janssen A.B.G., Kuiper J.J., Mooij W.M. & Trolle D. (2016) FABM-PCLake - linking aquatic ecology with hydrodynamics. *Geoscientific Model Development,* **9,** 2271-2278.

Jeppesen E., Søndergaard M., Søndergaard M. & Christoffersen K. (1998) The Structuring Role of Submerged Macrophytes in Lakes. In: *Ecological Series*. Springer Verlag, New York.

Jones J.I. & Sayer C.D. (2003) Does the fish-invertebrate-periphyton cascade precipitate plant loss in shallow lakes? *Ecology,* **84,** 2155-2167.

Kornijów R., Measey G.J. & Moss B. (2016) The structure of the littoral: effects of waterlily density and perch predation on sediment and plant-associated macroinvertebrate communities. *Freshwater Biology,* **61,** 32-50.

Matsuzaki S.S., Usio N., Takamura N. & Washitani I. (2009) Contrasting impacts of invasive engineers on freshwater ecosystems: an experiment and meta-analysis. *Oecologia,* **158,** 673-686.

Neal C., Hilton J., Wade A.J., Neal M. & Wickham H. (2006) Chlorophyll-a in the rivers of eastern England. *Science of the Total Environment,* **365,** 84-104.

Parker J.D., Burkepile D.E., Collins D.O., Kubanek J. & Hay M.E. (2007) Stream mosses as chemically-defended refugia for freshwater macroinvertebrates. *Oikos,* **116,** 302-312.

Pawar S., Dell A.I. & Savage V.M. (2012) Dimensionality of consumer search space drives trophic interaction strengths. *Nature,* **486,** 485-489.

Reynolds C.S. (1984) *The ecology of freshwater phytoplankton,* Cambridge University Press, Cambridge.

Reynolds C.S. (2000) Hydroecology of river plankton: the role of variability in channel flow. *Hydrological Processes*, **14,** 3119-3132.
Scheffer M., Hosper S.H., Meijer M.-L., Moss B. & Jeppesen E. (1993) Alternative equilibria in shallow lakes. *Trends in Ecology and Evolution*, **8,** 275-279.
Schriver P., Bægestrand J., Jeppesen E. & Søndergaard M. (1995) Impact of submerged macrophytes on fish-zooplankton phytoplankton interactions: large-scale enclosure experiments in a shallow eutrophic lake. *Freshwater Biology*, **33,** 255-270.
Soballe D. & Kimmel B. (1987) A large-scale comparison of factors influencing phytoplankton abundance in rivers, lakes, and impoundments. *Ecology*, **68,** 1943-1954.
Taniguchi H., Nakano S. & Tokeshi M. (2003) Influences of habitat complexity on the diversity and abundance of epiphytic invertebrates on plants. *Freshwater Biology*, **48,** 718-728.
Van Donk E. & Van De Bund W.J. (2002) Impact of submerged macrophytes including charophytes on phyto-and zooplankton communities: allelopathy versus other mechanisms. *Aquatic Botany*, **72,** 261-274.
Verhofstad M.J. & Bakker E.S. (2019) Classifying nuisance submerged vegetation depending on ecosystem services. *Limnology*, **20,** 55-68.

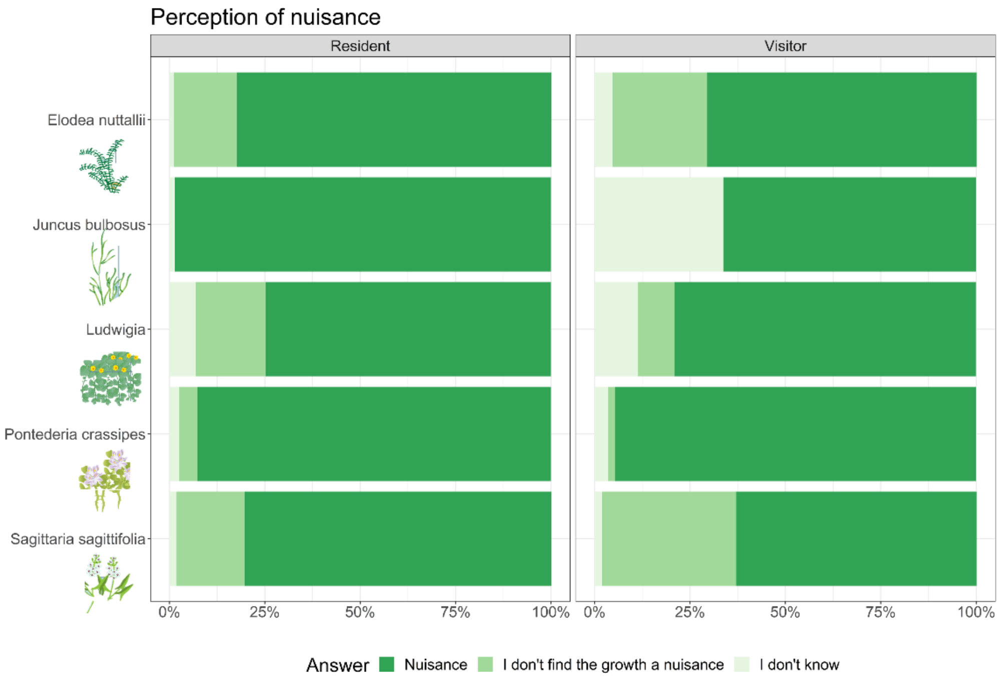

Figure S2. Fractions (%) of residents and visitors that have answered either that the one or more of the macrophyte growth levels were a nuisance, do not think it is a nuisance or do not know in each of the five cases. *Elodea nuttallii* was from Lake Kemnade, *Juncus bulbosus* from River Otra, *Ludwigia* spp. from Lake Grand-Lieu, *Pontederia crassipes* from Hartbeespoort Dam and *Sagittaria sagittifolia* from River Spree.

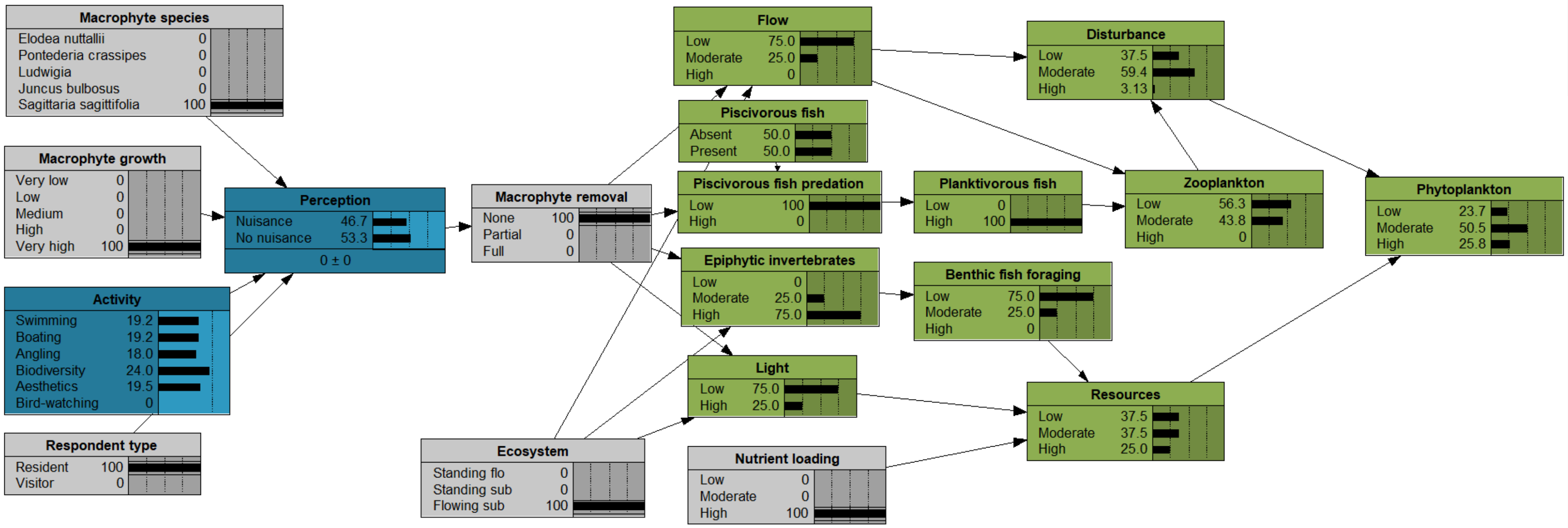

Figure S3. BN illustrating the probability of phytoplankton concentration for a riverine system with high nutrient loading given all the management practice is no removal of macrophytes. Grey boxes indicate nodes that have been specified.

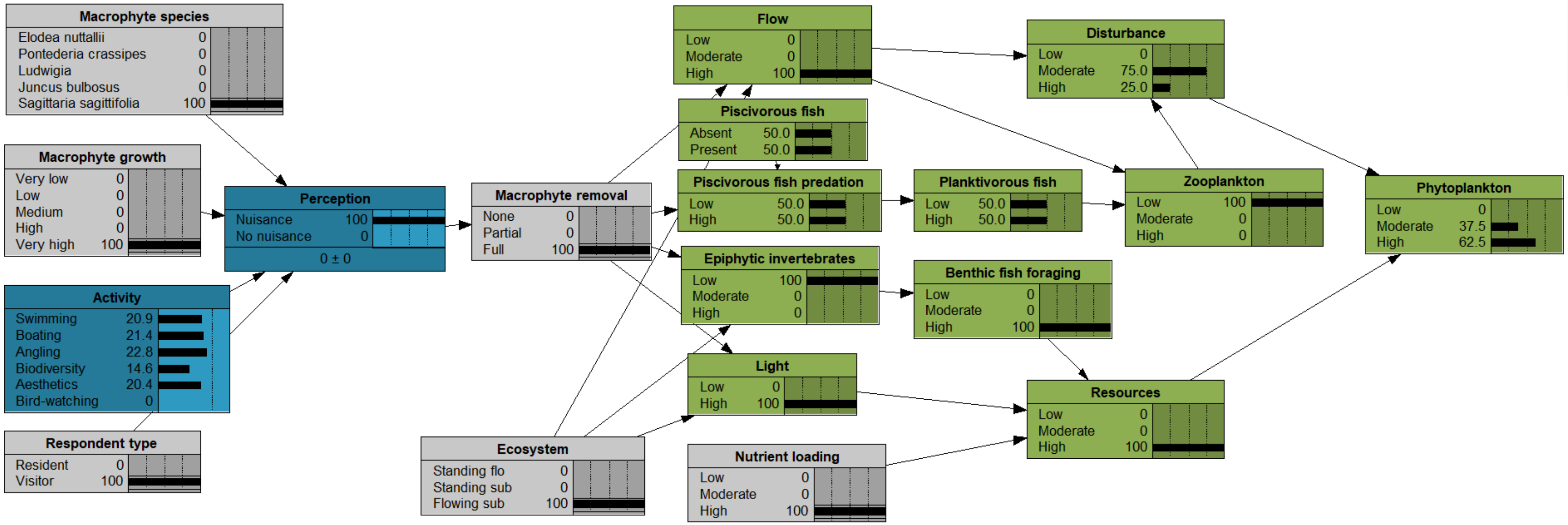

Figure S4. BN illustrating the probability of phytoplankton concentration for a riverine system with high nutrient loading given all the management practice is full removal of macrophytes. Grey boxes indicate nodes that have been specified.

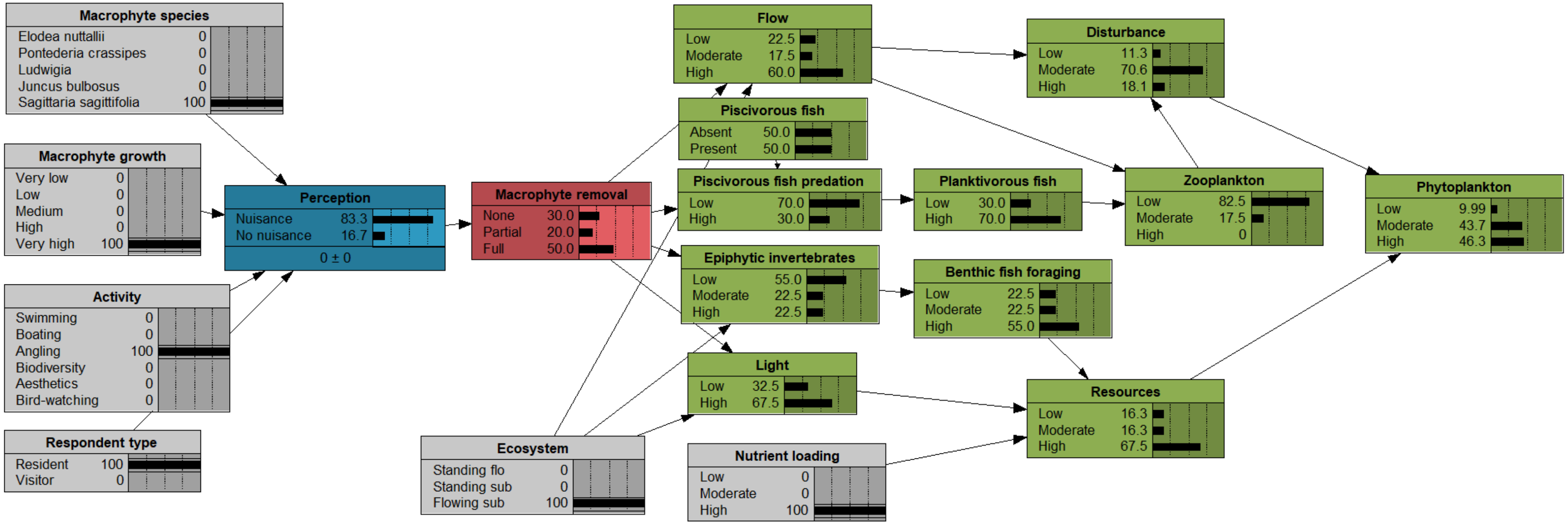

Figure S5. BN illustrating the probability of different management alternatives for a riverine system with high nutrient loading and very high *S. sagittifolia* growth given people using the river is only anglers. Grey boxes indicate nodes that have been specified.

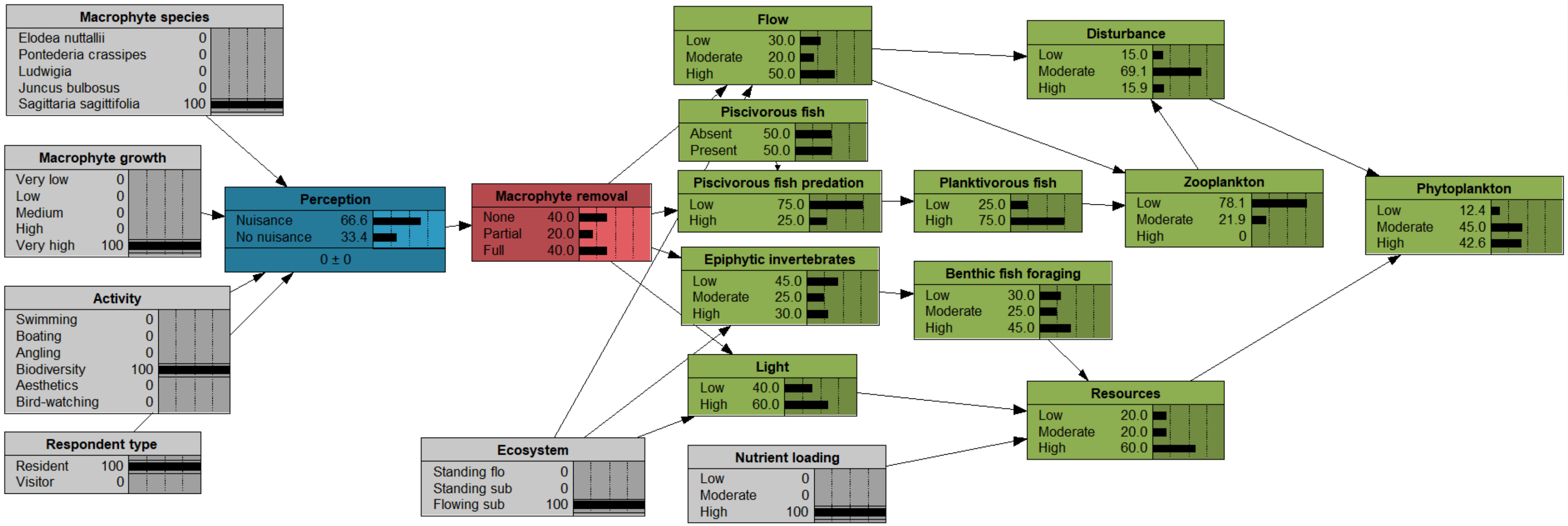

Figure S6. BN illustrating the probability of different management alternatives for a riverine system with high nutrient loading and very high *S. sagittifolia* growth given people using the river is appreciating biodiversity. Grey boxes indicate nodes that have been specified.

Supplementary Information III

Table S1. GLMMs results for probability of perceiving macrophyte as a nuisance in relation respondent type, macrophyte growth level and NEP-score. Df = degrees of freedom. Bold *P* values represent significant levels at 0.05.

	**Variable**	**Chisq**	**Df**	***P***
**Lake Kemnade**				
E. nuttalli				
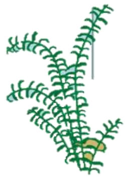	Respondent	10.63	1	**0.001**
	Macrophyte growth	218.22	1	**<0.001**
	NEP	0.00	1	0.980
**River Otra**				
J. bulbosus				
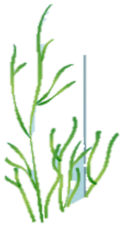	Respondent	21.25	1	**<0.001**
	Macrophyte growth	102.16	1	**<0.001**
	NEP	0.27	1	0.601
**Lake Grand-Lieu**				
Ludwigia spp.				
	Respondent	0.66	1	0.417
	Macrophyte growth	401.58	1	**<0.001**
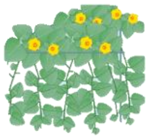	NEP	4.66	1	0.051
**Hartbeespoort Dam**				
P. crassipes				
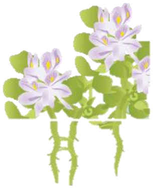	Respondent	0.51	1	0.477
	Macrophyte growth	173.21	1	**<0.001**
	NEP	1.43	1	0.232
	Macrophyte growth level:Respondent	9.19	1	**0.002**
**River Spree**				
S. sagittifolia				
	Respondent	4.38	1	**0.036**
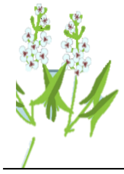	Macrophyte growth	110.18	1	**<0.001**
	NEP	2.83	1	0.093
	Macrophyte growth level:Respondent	9.96	1	**0.001**
