# Supplementary material for: Drivers of Perceived Nuisance Growth by Aquatic Plants
Source: Environ Manage. 2023 Jan 11;71(5):1024–36. doi: 10.1007/s00267-022-01781-x (PMC9832253; doi:10.1007/s00267-022-01781-x)
Supplement: Supplementary file 2 — Supporting Information I [file 267_2022_1781_MOESM2_ESM.pdf]

Supplementary Information I
Survey about Juncus bulbosus in River Otra

Dear survey participant,

Thank you for participating in this survey. It is part of a publicly funded research project called MadMacs. For more information on MadMacs, please see the last page of this questionnaire. Your answers will help us with estimating the environmental quality and issues important to the local community around the Otra river. We will ask you questions about your relationship to the Rysstad Basin, a part of the river Otra. All your answers are important – it is not necessary that you have specific knowledge on nature, water, tourism or the environment. There are no right and wrong answers.

Completing the survey takes about 10 minutes and will be anonymous. We follow the definitions of the General Data Protection Regulation (GDPR) for personal data of the European Union, which apply for data collection in Norway as well. If you have any questions regarding the study, please contact the interviewers (contact information on the last page).

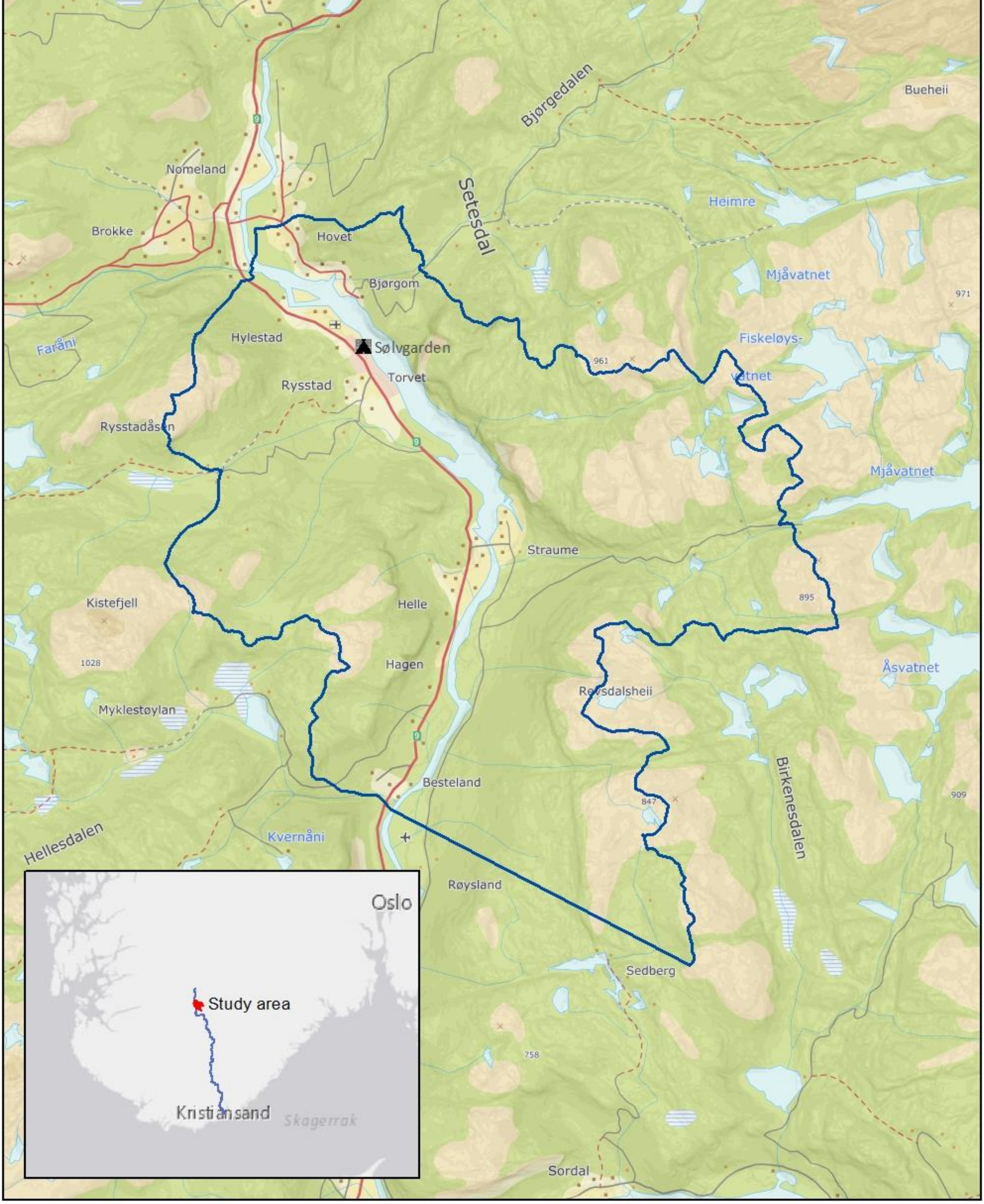

Rysstad basin

0 0.5 1 2 3 4 Kilometers

Study area

N 1:70,000

MADMACS

Service Layer Credits: Esri, HERE, Garmin, (c) OpenStreetMap contributors, and the GIS user community

Your relationship to the area

The map on the previous page shows the Rysstad Basin, which is our study area.

1. During the past 12 months, for what purpose have you been in the area? Multiple answers are possible.

a)	Short recreational visits (less than a day)	○
b)	Long recreational visits (more than a day)	○
c)	I live in the area	○
d)	I work in the area	○
e)	None of the above	○
f)	Other: ____________________________	○

If you did not recreate in the area during the last 12 months, please jump to question 6. Otherwise, continue to question 2.

2. **Please check the activities you typically do when visiting the area. Multiple answers are possible. Also indicate how many days you have done the activity in the last 12 months.**

		I do this in the area	Number of days in the last 12 months
a)	Walking/hiking	○	________________
b)	Running	○	________________
c)	Cycling/mountainbiking	○	________________
d)	Orienteering	○	________________
e)	Enjoying the river landscape	○	________________
f)	Photographing, painting or drawing nature	○	________________
g)	Berry/nut/mushroom gathering	○	________________
h)	Rock climbing	○	________________
i)	Motorbiking	○	________________
j)	Motorized boating	○	________________
k)	Rowing, sailing, canoeing	○	________________
l)	Hunting	○	________________
m)	Fishing	○	________________
n)	Swimming	○	________________
o)	Managing my property	○	________________
p)	Just relaxing	○	________________
q)	Other:______________________________	○	________________

3. Do you own property within 10km of the study area? Multiple answers are possible.

I own a house	I own a holiday home	I own farmland	I own forest	Other: ______________	I do not own property in the area
○	○	○	○	○	○

4. How far do you travel from your home to the part of the area where you recreate most often?

____________ km

5. How do you usually travel when recreating there? <u>Please mark only one option.</u>

a)	Car	○
b)	Public transport	○
c)	Bicycle	○
d)	Motorcycle	○
e)	Private boat	○
f)	Walking	○
g)	Motorhome/caravan	○
h)	Other: ____________________	○

6. **The Otra river, including the Rysstad Basin, generates benefits to society. Please indicate how important the following benefits are to your own wellbeing.**

		Very unimportant	Somewhat unimportant	Neither important, nor unimportant	Somewhat important	Very important	I don' t know
a)	Source of drinking water	○	○	○	○	○	○
b)	Possibilities for recreational fishing	○	○	○	○	○	○
c)	Clean water for nature	○	○	○	○	○	○
d)	Habitats for plants and animals	○	○	○	○	○	○
e)	Swimming possibilities	○	○	○	○	○	○
f)	Hydropower for electricity generation	○	○	○	○	○	○
g)	The fact that there is nature	○	○	○	○	○	○
h)	Educational possibilities	○	○	○	○	○	○
i)	Boating possibilities	○	○	○	○	○	○
j)	The beauty of the landscape	○	○	○	○	○	○
k)	Water storage to prevent floods	○	○	○	○	○	○

7. **Please indicate your top three most important benefits from the list above. Write down the letter from the first column related to the benefit.**

1. _______

2. _______

3. _______

Bulbous rush growth

This part of the survey is about your opinion on bulbous rush growth. Bulbous rush (*Juncus bulbosus L.*) is a water plant that develops very dense stands filling the water up to the surface in many slow flowing sections of the Otra river, including the Rysstad Basin. We use the term mass development for this. The plant is naturally occurring in shallow shore waters with fluctuating water level across Europe. In southwestern Norway it is removed in many places with a special mowing boat.

The following questions focus on the growth of bulbous rush in the Rysstad Basin and the different ways to manage this growth. We will focus on your perception and preferences on bulbous rush growth.

8. How do you personally perceive the current presence of bulbous rush in the Rysstad Basin? Please choose only one answer below.

Very negative	Negative	Neutral	Positive	Very positive	I don't know
○	○	○	○	○	○

9. The pictures below show different levels of bulbous rush growth in the Rysstad Basin. Image 1 shows the current level of bulbous rush growth. Please tick the pictures showing a level of growth you consider to be a nuisance, if any. You can select multiple pictures.

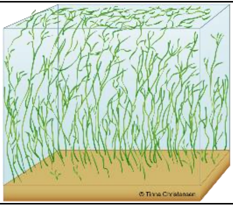	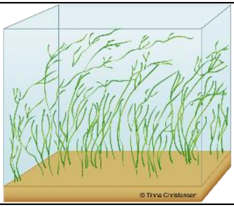	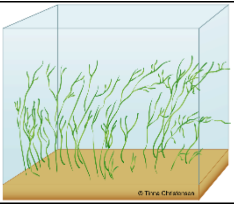	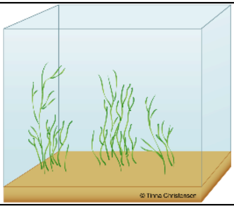	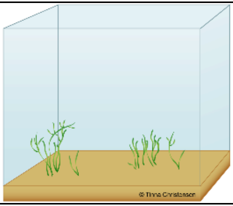
1. ○	2. ○	3. ○	4. ○	5. ○
I don't find the growth a nuisance. ○		I don't know. ○		

10. If you selected one or more of the above growth levels as a nuisance, what causes the nuisance? You can select multiple options.

a)	The plants make swimming less enjoyable	○
b)	The plants make it difficult for me to navigate a boat	○
c)	The plants make it harder for me to fish	○
d)	I am worried about the effect it might have on biodiversity	○
e)	I dislike the way the river looks with that many plants	○
f)	Other: ___________________________________	○
g)	I don't know	○

Currently, the bulbous rush in the Rysstad basin is removed in selected places used for recreation just before the main holiday season. This generally must be repeated every second year. We call this measure ‘selective local removal’. The photo below shows what this mowing looks like.

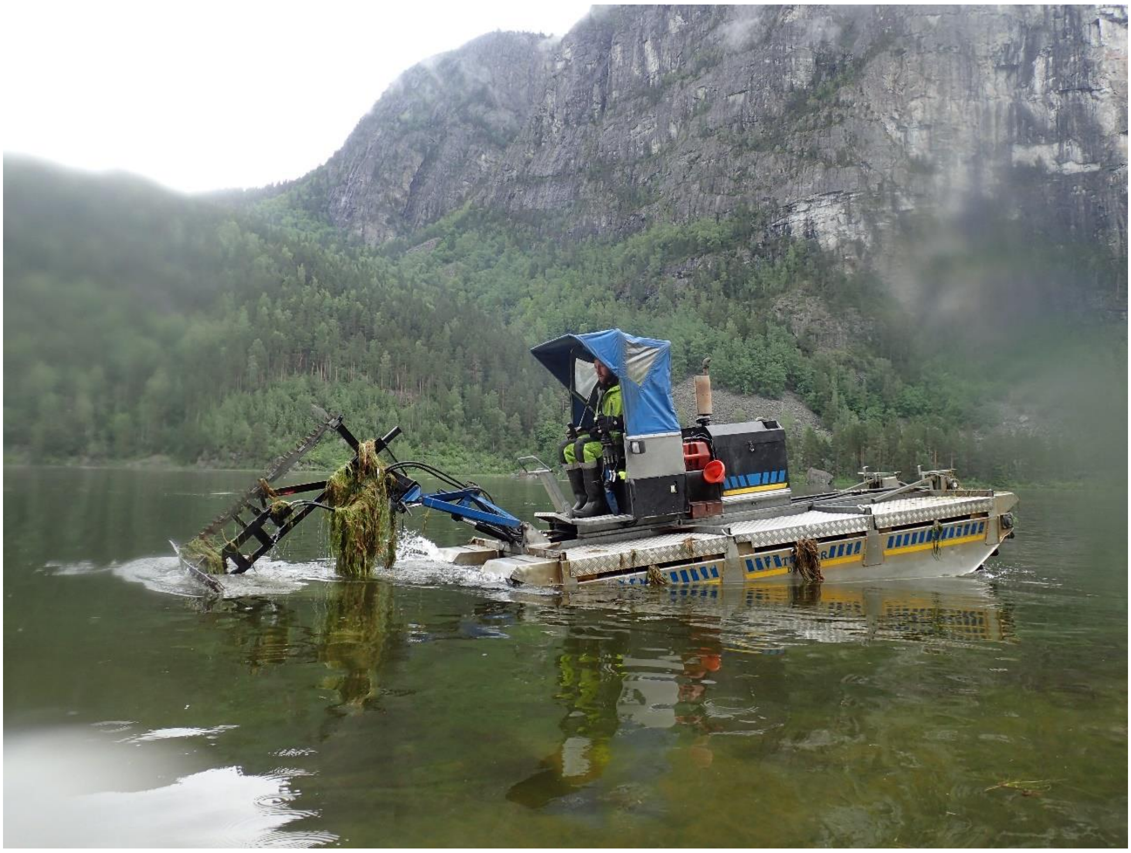

A new removal program funded by an increase in municipal household waste-water tax and tourist tax is possible, which will remove bulbous rush in the complete Rysstad basin instead of only in small locations. For residents, this will mean an increase in municipal household tax. For visitors, this tax will be paid once per year when visiting the area for at least one overnight stay. On the following pages, we will show you four different levels of removal. On each page, the level of growth without a new removal program is shown on the left side. Five characteristics of the river, for example possibilities for swimming and possibilities for boating are likewise shown.

11. **For each highlighted level, please consider how much you would be willing to pay per year in increased tax to reach that level as compared to the current situation. If you are a resident of the area, the municipal household waste-water tax applies. If you are a visitor, the individual tourist tax applies. Bear in mind that an increase in tax would reduce your income and consumption possibilities. <u>Please mark only one option.</u>**

	Current situation	With removal			
**Level of growth**	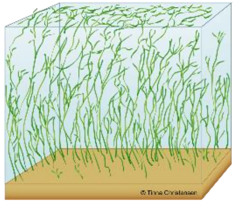	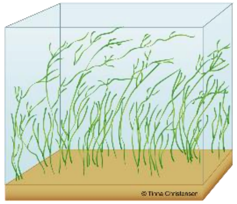			
**Possibilities for swimming**	Plants fill up the water and impede swimming.	There are visual plants up to the surface, continuously touching.	You feel plants below the surface most of the time, some tops are visible.	You occasionally feel plants below the surface.	Plants do not reach beyond the riverbed and you do not feel them.
**Possibilities for boating**	Rowing is hard. Engine rotors are very often clogged.	Plants slow down rowing and regularly clog engine rotors.	Plants brush up against boats and sometimes clog engine rotors.	Occasionally plants brush up against boats.	Rowing and motorized boating is unimpeded.
**Possibilities for fishing**	It's impossible to fish.	Lines often get stuck on plants on all depths.	Lines sometimes get stuck on plants on all depths.	Occasionally lines get stuck on plants when fishing near the bottom.	Fishing lines don't get stuck.
**Changes in biodiversity**	Low diversity in living environment for plants and animals.	Slightly diverse living environment for plants and animals.	Very diverse living environment for plants and animals.	Very diverse living environment for plants and animals.	Low diversity in living environment for plants and animals.
**Changes to the overall scenery**	Most of the river is filled up with plants.	Large beds are visible.	Some beds are visible up to the surface.	Some plant tops are visible in the water.	There are no visible plants in the water.

FOR RESIDENTS. For the highlighted level of removal, I am willing to pay the following increase in annual household tax:

0 kr.	50 kr.	100 kr.	250 kr.	500 kr.	1.000 kr.	2.500 kr.	5.000 kr.	More than 5.000 kr.	I don't know
○	○	○	○	○	○	○	○	○	○

FOR VISITORS. For the highlighted level of removal, I am willing to pay the following increase in individual tourist tax:

0 kr.	20 kr.	50 kr.	100 kr.	250 kr.	500 kr.	750 kr.	1.000 kr.	More than 1.000 kr.	I don't know
○	○	○	○	○	○	○	○	○	○

	Current situation	With removal			
**Level of growth**	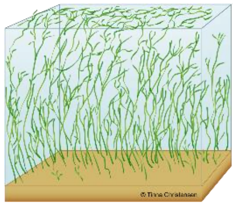		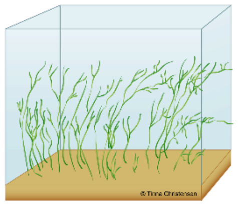		
**Possibilities for swimming**	Plants fill up the water and impede movement.	There are visual plants up to the surface, continuously touching.	You feel plants below the surface most of the time, some tops are visible.	You occasionally feel plants below the surface.	Plants do not reach beyond the riverbed and you do not feel them.
**Possibilities for boating**	Rowing is hard. Engine rotors are very often clogged.	Plants slow down rowing and regularly clog engine rotors.	Plants brush up against boats and sometimes clog engine rotors.	Occasionally plants brush up against boats.	Rowing and motorized boating is unimpeded.
**Possibilities for fishing**	It's impossible to fish.	Lines often get stuck on plants on all depths.	Lines sometimes get stuck on plants on all depths.	Occasionally lines get stuck on plants when fishing near the bottom.	Fishing lines don't get stuck.
**Changes in biodiversity**	Low diversity in living environment for plants and animals.	Slightly diverse living environment for plants and animals.	Very diverse living environment for plants and animals.	Very diverse living environment for plants and animals.	Low diversity in living environment for plants and animals.
**Changes to the overall scenery**	Most of the river is filled up with plants.	Large beds are visible.	Some beds are visible up to the surface.	Some plant tops are visible in the water.	There are no visible plants in the water.

FOR RESIDENTS. For the highlighted level of removal, I am willing to pay the following increase in annual household tax:

0 kr.	50 kr.	100 kr.	250 kr.	500 kr.	1.000 kr.	2.500 kr.	5.000 kr.	More than 5.000 kr.	I don't know
○	○	○	○	○	○	○	○	○	○

FOR VISITORS. For the highlighted level of removal, I am willing to pay the following increase in individual tourist tax:

0 kr.	20 kr.	50 kr.	100 kr.	250 kr.	500 kr.	750 kr.	1.000 kr.	More than 1.000 kr.	I don't know
○	○	○	○	○	○	○	○	○	○

	Current situation	With removal			
**Level of growth**	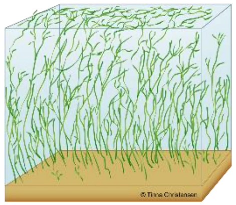			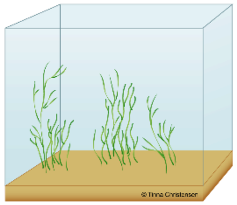	
**Possibilities for swimming**	Plants fill up the water and impede movement.	There are visual plants up to the surface, continuously touching.	You feel plants below the surface most of the time, some tops are visible.	You occasionally feel plants below the surface.	Plants do not reach beyond the riverbed and you do not feel them.
**Possibilities for boating**	Rowing is hard. Engine rotors are very often clogged.	Plants slow down rowing and regularly clog engine rotors.	Plants brush up against boats and sometimes clog engine rotors.	Occasionally plants brush up against boats.	Rowing and motorized boating is unimpeded.
**Possibilities for fishing**	It's impossible to fish.	Lines often get stuck on plants on all depths.	Lines sometimes get stuck on plants on all depths.	Occasionally lines get stuck on plants when fishing near the bottom.	Fishing lines don't get stuck.
**Changes in biodiversity**	Low diversity in living environment for plants and animals.	Slightly diverse living environment for plants and animals.	Very diverse living environment for plants and animals.	Very diverse living environment for plants and animals.	Low diversity in living environment for plants and animals.
**Changes to the overall scenery**	Most of the river is filled up with plants.	Large beds are visible.	Some beds are visible up to the surface.	Some plant tops are visible in the water.	There are no visible plants in the water.

FOR RESIDENTS. For the highlighted level of removal, I am willing to pay the following increase in annual household tax:

0 kr.	50 kr.	100 kr.	250 kr.	500 kr.	1.000 kr.	2.500 kr.	5.000 kr.	More than 5.000 kr.	I don't know
○	○	○	○	○	○	○	○	○	○

FOR VISITORS. For the highlighted level of removal, I am willing to pay the following increase in individual tourist tax:

0 kr.	20 kr.	50 kr.	100 kr.	250 kr.	500 kr.	750 kr.	1.000 kr.	More than 1.000 kr.	I don't know
○	○	○	○	○	○	○	○	○	○

	Current situation	With removal			
**Level of growth**	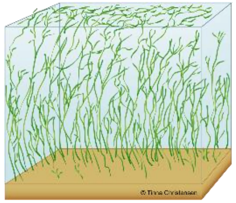				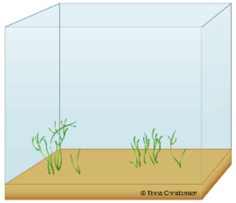
**Possibilities for swimming**	Plants fill up the water and impede movement.	There are visual plants up to the surface, continuously touching.	You feel plants below the surface most of the time, some tops are visible.	You occasionally feel plants below the surface.	Plants do not reach beyond the riverbed and you do not feel them.
**Possibilities for boating**	Rowing is hard. Engine rotors are very often clogged.	Plants slow down rowing and regularly clog engine rotors.	Plants brush up against boats and sometimes clog engine rotors.	Occasionally plants brush up against boats.	Rowing and motorized boating is unimpeded.
**Possibilities for fishing**	It's impossible to fish.	Lines often get stuck on plants on all depths.	Lines sometimes get stuck on plants on all depths.	Occasionally lines get stuck on plants when fishing near the bottom.	Fishing lines don't get stuck.
**Changes in biodiversity**	Low diversity in living environment for plants and animals.	Slightly diverse living environment for plants and animals.	Very diverse living environment for plants and animals.	Very diverse living environment for plants and animals.	Low diversity in living environment for plants and animals.
**Changes to the overall scenery**	Most of the river is filled up with plants.	Large beds are visible.	Some beds are visible up to the surface.	Some plant tops are visible in the water.	There are no visible plants in the water.

FOR RESIDENTS. For the highlighted level of removal, I am willing to pay the following increase in annual household tax:

0 kr.	50 kr.	100 kr.	250 kr.	500 kr.	1.000 kr.	2.500 kr.	5.000 kr.	More than 5.000 kr.	I don't know
○	○	○	○	○	○	○	○	○	○

FOR VISITORS. For the highlighted level of removal, I am willing to pay the following increase in individual tourist tax:

0 kr.	20 kr.	50 kr.	100 kr.	250 kr.	500 kr.	750 kr.	1.000 kr.	More than 1.000 kr.	I don't know
○	○	○	○	○	○	○	○	○	○

12. Some of the five characteristics that will change due to an increase in removal might be more important to you than others. Please distribute 100 points over the different characteristics according to the importance they had when making your choices in the previous question.

a)	Possibilities for swimming	____ / 100
b)	Possibilities for boating	____ / 100
c)	Possibilities for fishing	____ / 100
d)	Changes to biodiversity	____ / 100
e)	Changes to aesthetics	____ / 100
f)	Total	100 / 100

13. Are there any other aspects, either negative or positive, about bulbous rush that you considered when deciding on your willingness to pay? If so, write them down here:

____

14. How certain are you about the amount you want to pay for bulbous rush removal?

Very uncertain	Slightly uncertain	Slightly certain	Very certain	I don’t know
○	○	○	○	

15. How realistic do you find the suggested costs for bulbous rush removal?

Very unrealistic	Slightly unrealistic	Slightly realistic	Very realistic	I don’t know
○	○	○	○	○

16. Please rate to what degree you agree with the following statement:
I believe that tax collected for a bulbous rush removal program will be efficiently spent.

Strongly disagree	Disagree	Agree	Strongly agree	I don't know
○	○	○	○	○

17. If you chose no increase of tax, please indicate why you did so. <u>If not, you can skip this question.</u>

a)	I prefer to let the bulbous rush grow naturally without removing it	○
b)	I did not find an increase of tax for removal realistic	○
c)	I do not want to pay an extra tax on principle	○
d)	I do not have enough money to pay an extra tax	○
e)	I believe the money would end up in the wrong hands	○
f)	I believe we should spend money on solving the causes instead of fighting the symptoms	○
g)	Other: ________________________________	○

Background information

18. What is your age?

____

19. What is your gender?

a)	Male	○
b)	Female	○

20. What is your nationality?

a)	Norwegian	○
b)	Other: __________________	○

21. In what kind of neighbourhood did you grow up?

a)	Rural area or village	○
b)	City, town or urban agglomeration	○

22. In what kind of neighbourhood do you currently live?

a)	Rural area or village	○
b)	City, town or urban agglomeration	○

23. What is the highest level of education you have received? <u>Please choose only one answer.</u>

a)	Primary school	○
b)	Secondary school	○
c)	Vocationary education	○
d)	University degree	○
e)	Other education: _________________________	○

24. Are you currently employed? <u>Please choose only one answer.</u>

a)	Yes, I am employed	○
b)	I am unemployed	○
c)	I am retired	○
d)	I am a student	○
e)	I manage the household	○
f)	No, other reason	○

25. In which sector do you work (pensioners and unemployed: past work, students: future work)? Please only choose one answer.

		Public sector	Private sector
a)	Agriculture	○	○
b)	Forestry	○	○
c)	Building and construction	○	○
d)	Manufacturing industry	○	○
e)	Energy and mining	○	○
f)	Fishery	○	○
h)	Healthcare	○	○
i)	Education	○	○
j)	Other services (anything not producing material goods)	○	○
k)	Other:___________________	○	○

26. What is your monthly gross income level in NOK?

a)	No income	○
b)	Less than 15 000 kr.	○
c)	15 000 – 19 999 kr.	○
d)	20 000 – 24 999 kr.	○
e)	25 000 – 29 999 kr.	○
f)	30 000 – 39 999 kr.	○
g)	40 000 – 59 999 kr.	○
h)	60 000 – 79 999 kr.	○
i)	80 000 – 99 999 kr.	○
j)	over 100 000 kr.	○
k)	I do not want to disclose my income	○

27. In what type of household do you live?

a)	Single	○
b)	Couple	○
c)	Couple with underaged children	○
d)	Other adult household (all over 18 yrs.)	○
e)	Other	○

28. Please indicate to what extent do you agree or disagree with the following statements in general.

		Strongly disagree	Disagree	Agree	Strongly agree	I don' t know
a)	We are approaching the limit of the number of people the earth can support	○	○	○	○	○
b)	Humans have the right to modify the natural environment to suit their needs	○	○	○	○	○
c)	When humans interfere with nature it often produces disastrous consequences	○	○	○	○	○
d)	Human ingenuity will insure that we do NOT make the earth unlivable	○	○	○	○	○
e)	Humans are severely abusing the environment	○	○	○	○	○
f)	The earth has plenty of natural resources if we just learn how to develop them	○	○	○	○	○
g)	Plants and animals have as much right as humans to exist	○	○	○	○	○
h)	The balance of nature is strong enough to cope with the impacts of modern industrial nations	○	○	○	○	○
i)	Despite our special abilities humans are still subject to the laws of nature	○	○	○	○	○
j)	The so–called ''ecological crisis'' facing humankind has been greatly exaggerated	○	○	○	○	○

		Strongly disagree	Disagree	Agree	Strongly agree	I don’ t know
k)	The earth is like a spaceship with very limited room and resources	○	○	○	○	○
l)	Humans were meant to rule over the rest of nature	○	○	○	○	○
m)	The balance of nature is very delicate and easily upset	○	○	○	○	○
n)	Humans will eventually learn enough about how nature works to be able to control it	○	○	○	○	○
o)	If things continue on their present course, we will soon experience a major ecological catastrophe	○	○	○	○	○

Thank you very much for taking the time to fill in this questionnaire. Below there is some room for additional comments. The final page gives some additional information on our research project.

MADMACS

About MadMacs

MadMacs (**Ma**ss **d**evelopment of aquatic **ma**crophyte**s**) is an international research project funded by Water JPI (www.waterjpi.eu) together with local partners (Krypsivprosjektet på Sørlandet) and is a collaboration between the following research institutes and universities:

Norwegian Institute for Water Research, NIVA (Norway)
Norwegian University of Life Sciences, NMBU (Norway)
Rhodes University (South Africa)
Leibniz-Institute of Freshwater Ecology and Inland Fisheries, IGB (Germany)
University of Rennes, UMR ECOBIO (France)
Universidade Federal do Paraná, UFPR (Brazil)

Mass development of aquatic macrophytes (water plants) in rivers and lakes is today considered a worldwide problem and considerable resources are spent on macrophyte removal each year. In MadMacs, our objective is to evaluate the causes and consequences of macrophyte removal on ecosystem structure, functions and services and to provide consistent and comparable data from five countries across three continents. Our research will contribute to improved management of aquatic ecosystems. This survey is developed by Kirstine Thiemer, PhD candidate at NIVA/NMBU and Bart Immerzeel, PhD candidate at NMBU. Our aim is to estimate the benefits and disadvantages society derives from macrophytes, and to examine if these estimates change when macrophytes are removed. If you have any further questions, please contact: kirstine.thiemer@niva.no or bart.immerzeel@nmbu.no

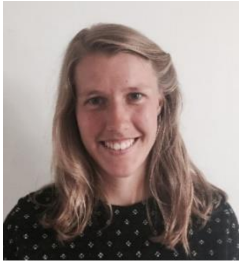

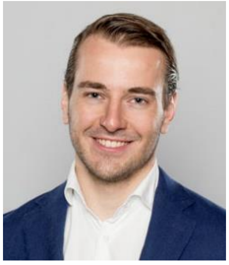

For more information on MadMacs, please have a look at our website by scanning the QR code below or by going to: https://www.niva.no/en/projectweb/madmacs
